# Microcystin congeners in Lake Erie follow the seasonal pattern of nitrogen availability

**DOI:** 10.1016/j.hal.2023.102466

**Published:** 2023-06-02

**Authors:** Justin D. Chaffin, Judy A. Westrick, Laura A. Reitz, Thomas B. Bridgeman

**Affiliations:** a F.T. Stone Laboratory and Ohio Sea Grant, The Ohio State University, 878 Bayview Ave. P.O. Box 119, Put-In-Bay, OH 43456, USA; b Lumigen Instrument Center, Wayne State University, 5101 Cass Ave, Detroit, MI 48202, USA; c Department of Biological Sciences, Bowling Green State University, Life Sciences Building, Bowling Green, OH 43402, USA; d Lake Erie Center, University of Toledo, Oregon, OH, 43416, USA

**Keywords:** Cyanobacteria, Eutrophication, Microcystis, Toxicity

## Abstract

Cyanobacteria harmful algal blooms produce many toxic secondary metabolites called cyanotoxins. The most studied group of cyanotoxins are microcystins (MC), with over 300 congeners reported. MC-LR is the most studied congener because of its abundance and toxicity. Recent toxicology studies suggest that more hydrophobic MC congeners such as MC-LA, MC-LF, and MC-LW may be less abundant but up to seven times more toxic than MC-LR, whereas, MC-RR’s toxicity is only one-fifth that of MC-LR. Hence, understanding the environmental stressors that change the MC congener profile is critical to assessing the negative impact on environmental and human health. A two-year field and experimental study investigated seasonal and spatial changes of MC congener profiles in the western basin of Lake Erie. Both studies showed that nitrogen enrichment favored the production of nitrogen-rich MC-RR (C_49_H_75_N_13_O_12_). The field study showed that nitrogen depletion favored the low-nitrogen MC-LA (C_46_H_67_N_7_O_12_). MC-LR (a medium N level, C_49_H_75_N_10_O_12_) accounted for ~30% to 50% of the total MC concentration and was stable across nitrogen concentrations. Using the relative toxicity and concentrations of each MC congener, both LC-MS/MS and ELISA overestimated the toxicity early bloom (July) and underestimated it late bloom (September). On 24 July 2019, highly toxic MC-LW and MC-LF were detected at nearshore stations with relative toxicity exceeding drinking water standards. This study demonstrated that the less toxic, high nitrogen MC-RR dominated under nitrogen-replete conditions in the early season, whereas the more toxic, less nitrogen MC-LA dominated under nitrogen-limited conditions later in the season.

## 1. Introduction

Cyanobacterial blooms have become a global problem due to excessive nutrient loading and a warming climate (Paerl and Huisman, 2008). Cyanobacterial blooms are problematic in part due to their production of secondary metabolites that have toxic effects, often termed “cyanotoxins” (Buratti et al., 2017; Carmichael, 1992). Microcystins (MCs) are among the most frequently encountered cyanotoxins, occur in the highest concentrations, and are a highly potent hepatotoxin (Harke et al., 2016b; Loftin et al., 2016). Understanding triggers of MC congener production are of paramount importance for developing toxicity forecasts and protecting human health.

Microcystins are a class of cyanotoxins with over 300 known MC congeners (Spoof and Catherine, 2017). Microcystin congeners are identified and named based on two variable amino acids at positions 2 and 4 of the MC molecule. For example, microcystin-LR has leucine (L) and arginine (R) and microcystin-LA has leucine and alanine (A) in position 2 and 4, respectively. Furthermore, changes in these amino acids alter chemical and physical molecular characteristics and hence may dramatically influence the toxicity of each MC congener. Despite the high number of congeners, all health recommendations and guidelines are based on the toxicity of MC-LR because early studies focused on MC-LR (Bishop et al., 1959; Fawell et al., 1999; MacKintosh et al., 1990). The World Health Organization recently added short-term drinking water guidelines of 12 μg/L for adults and 3 μg/L for small children, yet maintaining a lifetime guideline of 1 μg/L (World Health Organization, 2020). The United States Environmental Protection Agency supports a more stringent drinking water guideline of 1.6 and 0.3 μg/L for people over and under school-age children, respectively (US EPA, 2019). More recent toxicological studies have investigated other common MCs and reported the toxicity of other MCs relative to that of MC-LR (values less than 1.0 being less toxic than MC-LR and values greater than 1.0 being more toxic). Combined, these studies report nearly a two order of magnitude relative toxicity range from 0.08 to 0.3 for MC-RR (Chernoff et al., 2021; Faassen and Lürling, 2013; Gupta et al., 2003) to 7.0 for MC-LW and MC-LF) (Faassen and Lürling, 2013; Fischer et al., 2010). Furthermore, water samples collected from lakes during cyanobacterial blooms often contain a mixture of congeners (Díez-Quijada et al., 2019; Dyble et al., 2008; Palagama et al., 2020). Therefore, the overall toxicity of a water sample is based on the concentration of the individual congeners.

The most common method to detect and quantify MCs in water samples is an enzyme-linked immunosorbent assay (ELISA). The ELISA method is congener-independent, and concentrations are reported as “total MC μg/L, or parts per billion” without information on the congener profile. Microcystin-LR is used as the standard to generate the antibody-based calibration curve, but different congeners within the sample do not have the same reactivity to the antibody as MC-LR (Fischer et al., 2001; He et al., 2017). For example, MC-RR and MC-YR have a reactivity relative to MC-LR of 0.5 and 1.67, respectively (Fischer et al., 2001). Therefore, the “total MC” reported by ELISA would more accurately be described as MC-LR concentration equivalents. Finally, ELISA does not give congener information, and each congener toxicity varies, resulting in a high likelihood that the toxicity of the sample is either over or underestimated. The liquid chromatography mass spectrometry method for MCs overcomes many issues with ELISA as it quantifies the concentration of individual congeners. However, because the ELISA analysis is less expensive and can be performed on site, the ELISA is more commonly used by water practitioners.

Nitrogen (N) accounts for 9.7% to 18.0% of the MC molecular mass or a carbon-to N ratio of 4.9 to 7.4, depending on the congener (Table 1). Colonies of *Microcystis*, a common MC producer (Harke et al., 2016b), are 7% N by mass (Chaffin et al., 2011), therefore, MCs are N-rich compounds. Due to this stoichiometry, many field studies (Horst et al., 2014; Van de Waal et al., 2014) and laboratory studies (Harke and Gobler, 2013; Jankowiak et al., 2019; Wagner et al., 2019) have shown that low N availability decreases total MC cellular quota. However, the few laboratory studies that have investigated the drivers of MCs at the congener level used specific lab cultures strains (Puddick et al., 2016; Tonk et al., 2008; Wagner et al., 2021) that omit interactions with the phycosphere and co-occurring strains. Many field studies that reported MC congeners have been mostly descriptive (Dyble et al., 2008; Palagama et al., 2020; Taranu et al., 2019). Van de Waal et al. (2009) conducted chemostat experiments with *Microcystis* and collected data from 12 lakes dominated by *Microcystis*. Their results showed that MC-RR, an N-rich congener, became more abundant under conditions of high N availability and low cellular carbon-to-N ratios. However, Van de Waal et al. (2009) only analyzed for three MC congeners (MC-LR, -RR, and -YR). This study included both field and experimental data to investigate more comprehensive MC congener profile trends in natural *Microcystis* blooms, as well as what drives the MC congener profile in Lake Erie.

Since the late 1990s, the western basin of Lake Erie has had annual summertime cyanobacterial blooms dominated by the MC-producer *Microcystis aeruginosa* (Bridgeman et al., 2013; Ho et al., 2017). While other cyanobacteria are present in the open waters of the western basin, *Microcystis* is usually the main MC producer (Ouellette et al., 2006), but *Planktothrix* can be common in the nearshore zones (Jankowiak et al., 2019). Phytoplankton growth and MC production transition from phosphorus (P)-limited to N-limited throughout the summer as N availability declines in Lake Erie (Chaffin et al., 2018; Jankowiak et al., 2019). A biweekly survey of eight routine monitoring locations in the western basin during the 2016 and 2017 bloom seasons found 26 different MC congeners with the most prevalent congeners being MC-LR, -RR, - LA, and -YR (Palagama et al., 2020). Our study objectives were to determine how congener concentration correlates with environmental parameters and to estimate the total relative toxicity of each sample. We approached these objectives with a two-year study that included both a spatial-temporal field survey and laboratory experiments. We hypothesized that N-rich MC congeners (e.g., MC-RR) would be more present in early summer (ex: July) when ambient N is available and that N-low congeners (e.g., MC-LA) would be more abundant in late summer and fall (ex: September) when ambient N concentrations are lower.

## 2. Methods and supplies

### 2.1. Routine monitoring

**Fig. 1.**
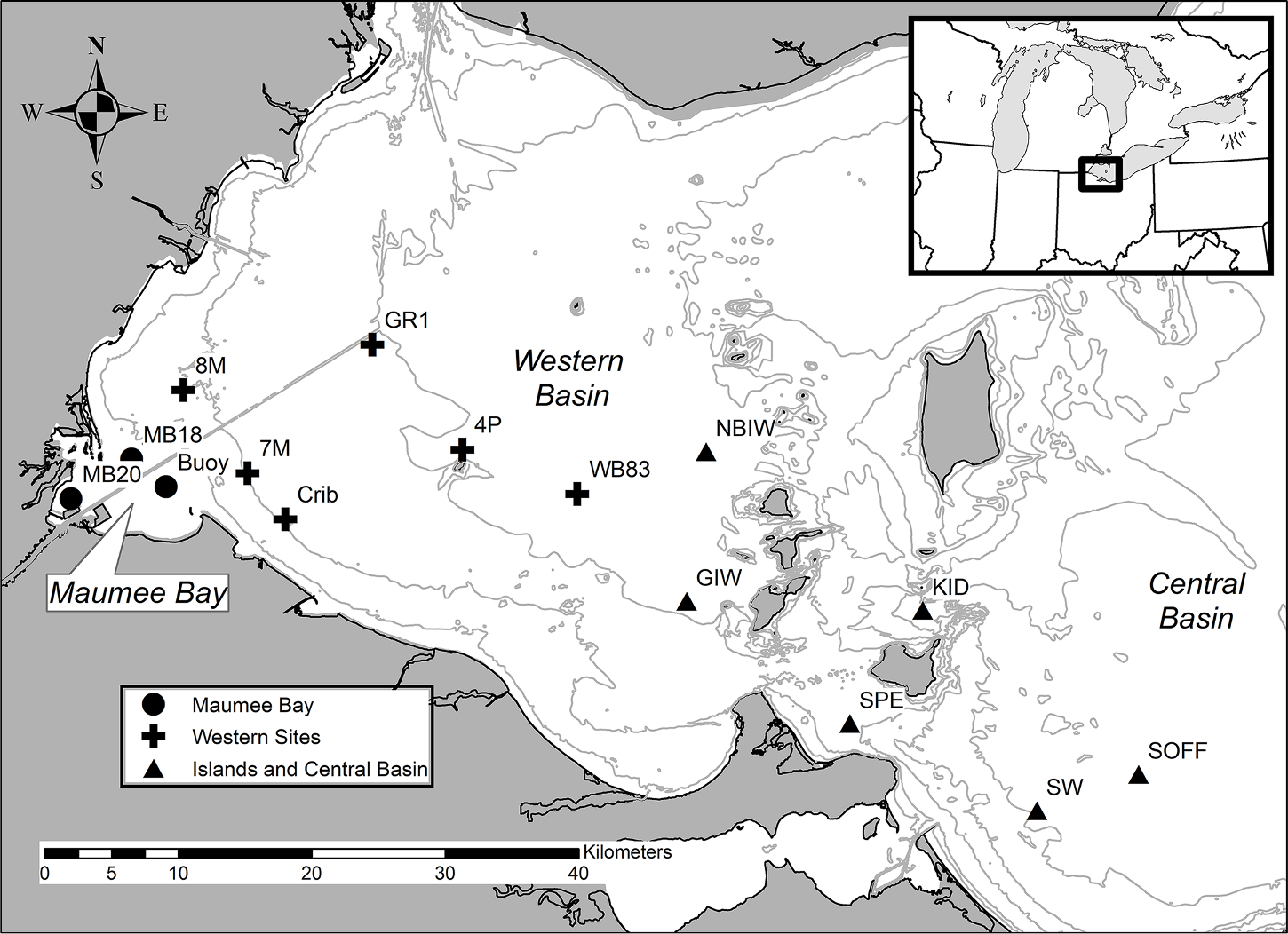
Map of sample locations in western Lake Erie. The Maumee Bay sites are indicated by circles, the Western sites by crosses, and the near Islands and Central Basin sites as triangles. Contour lines are 3, 5, 8, 10, and 12 m depth.

Fifteen sites in Lake Erie were sampled at weekly to biweekly intervals from June through September during 2018 and 2019. These sites have been monitored by the University of Toledo and Ohio State University since the early 2000s (Conroy et al., 2005; Moorhead et al., 2008). The sites ranged from 1.7 km to 82.7 km from the mouth of the Maumee River (the main source of nutrients to Lake Erie (Scavia et al., 2016)) and depths from 2 m to 15 m (Fig. 1). The eight sites closest to the Maumee River were also sampled by Palagama et al. (2020) during the two years before our study.

Physicochemical parameters (pH, conductivity, temperature, dissolved oxygen, turbidity) were recorded at 1-meter intervals from surface to bottom with an YSI EXO2 sonde (YSI Inc., Yellow Springs, OH, USA). Grab samples were collected with an integrated water column sampler from the surface to 1 meter above the lake bottom or to the thermocline (or up to 8 m maximum depth) and the water was deposited into a clean and pre-rinsed 20 L bucket (Golnick et al., 2016). Lake water from the bucket was poured into a dark 1 L or 2 L polycarbonate (PC) bottles for chlorophyll analysis, 250 mL polyethylene terephthalate glycol (PETG) or PC for nutrient analysis, a 1 L glass jar with 1% Lugol’s solution for phytoplankton enumeration, and 60 mL amber glass vial for MCs (analytical methods below). Water (100 mL) from the PETG or PC bottles were 0.45 μm-filtered upon collection for analysis for dissolved nutrients and stored in a 60 mL PETG bottle, and the remaining sample volume was saved for analysis of total P and N. All samples were stored in a dark cooler on ice while being transported back to the laboratory. Water for chlorophyll analysis was processed upon arriving at the laboratory. Samples for nutrients and MCs were frozen (−20 °C) until analysis.

### 2.2. Experiments

Surface lake water (40 L) was collected twice monthly from sites MB18 and WB83 for nutrient enrichment bioassays to determine the impact of N form on MC congeners. These two sites were selected for experimentation due to differing water quality, but both being prone to cyanobacterial blooms. Complete methods for these experiments are presented in Chaffin et al. (2022). Briefly, there were four nutrient enrichment treatments: (1) no enrichment control, (2) phosphate and nitrate, (3) phosphate and ammonium, and (4) phosphate and urea. All enrichments were 1 μmol/L P and 100 μmol/L N (50 μmol/L urea = 100 μmol/L N). Since previous studies have shown that Lake Erie *Microcystis* blooms reach the highest biomass and produce the most MCs when both P and N were added (Chaffin et al., 2018; Harke et al., 2016a; Jankowiak et al., 2019), the primary limiting nutrient (P or N) was not a concern. Both P and three forms of N to ensure we saw a response from the *Microcystis*., and to investigate if reduced forms of N (ammonium, urea) would result in more N-rich MCs than nitrate. Hence, acknowledging that the difference between the control and the P and N enrichments could be due to either the P or the N, and inferring that the differences among the P and N enrichments were due to the N form. All treatments were replicated with three separate 2.4 L PETG clear bottles. Bottles were incubated for 72 h in limnocorrals in Lake Erie off docks at the Lake Erie Center (for MB18 water) and Stone Laboratory (for WB83 water). Bottles were sampled at hours 0 and 72 for analysis of chlorophyll and particulate MCs (analytical methods below).

### 2.3. Analytical methods

Concentrations of nitrate, nitrite, ammonium, and dissolved reactive phosphate were quantified in the 0.45 μm-filtered samples and total P and total Kjdelhal N (TKN) on unfiltered samples following standard EPA methods (as in Chaffin et al. (2021). Concentrations of nitrate, nitrite, and TKN were summed to calculate total N concentration. Concentrations of nitrate, nitrite, and ammonium were summed to calculate dissolved inorganic N (DIN).

A bbe Moldanke FluoroProbe with a bench-top cuvette reader measured the cyanobacteria-specific chlorophyll (chl) *a* concentrations (Bridgeman et al., 2012). The cyanobacteria-specific chl *a* concentrations were normalized to the chl concentrations measured by traditional filter-extraction methods (Bridgeman et al., 2012). Phytoplankton biovolume from the Lugol’s preserved sample (from WB83) or from a 2% formalin-preserved sample (from MB18 initial samples) was quantified using a FlowCam (Hrycik et al., 2019) at 100x magnification (v. 8400, supplemental document for more information).

Total MCs from lake samples were measured in whole water samples, whereas particulate MC from the experiments were determined by extracting MC captured on a filter. We opted for particulate MCs for the experiments to limit our analysis of newly produced MCs. For particulate MCs, 50 to 100 mL, depending on the biomass, were filtered onto 1.2 μm polycarbonate filters. The filters were placed in 10 mL of deionized water in an amber glass vial that was then frozen (−20 °C). From this point on, both total and particulate MCs were processed following the same protocol. Microcystins were extracted from cells using Ohio EPA’s protocol of three freeze/thaw cycles, and then the lysate was filtered through a GMF filter (glass microfiber filter, 0.45 μm) into an amber glass vial and frozen at −20 °C until analysis (Ohio EPA, 2018). Liquid chromatography with tandem mass spectrometry (LC-MS/MS) quantified 12 MC congeners ([D-A*sp*^3^]-MC-RR, MC-RR, MC-YR, MC–HtyR, MC-LR, [D-Asp^3^]-MC-LR, MC–HilR, MC-WR, MC-LA, MC-LY, MC-LW, and MC-LF (Birbeck et al. 2019b). The detection limit varied between congeners with a range of < 0.5 to 5.0 part per trillion. The ELISA method was also used to measure total MCs from the lake samples using Abraxis ELISA kits, following the Ohio EPA protocol on the freeze/thawed samples (Ohio EPA, 2018).

### 2.4. Data analysis

All 12 MC congeners were summed to calculate the total MC concentration, and the percentage of the total was calculated for each congener (abbreviated as %MC-XX). The data analysis was limited to samples with a total MC concentration of greater than 0.025 μg/L, which gave 168 samples. To facilitate data visualization among the 15 sites, we placed sample locations into three groups based on physicochemical variables and proximity to the Maumee River. The three sites within 10 km of the river mouth were termed “Maumee Bay,” the six sites between Maumee Bay and the Bass Islands were termed “Western sites,” and the remaining site sites near the Bass Islands and Kelleys Island and in the central basin were termed “Islands and Central Basin sites.” A 2-factor multivariate analysis of covariance (MANCOVA) was conducted to determine if sample site grouping, years (2018 and 2019), and the day of the year (as a covariate) affected the %MC-XX for each congener. The Pillai’s Trace statistic because Box’s test of equality of covariance matrices indicated that covariance matrices were not equal among all variables (*p* < 0.001). Then ANCOVAs were conducted because the MANCOVA showed significant effects for site and day of year (see results). Additionally, a Pearson correlation analysis was conducted to determine the relationship among environmental variables (nutrients, biomass, and total MC concentration) and the %MC-XX for each congener.

To determine the relative toxicity of each grab sample, the concentration of each congener was multiplied by the relative toxicity factor (Table 1). Toxicity factors (the LD_50_, no observed adverse effects level, lowest observed adverse effects level, EC_50_) were obtained from the literature and normalized to MC-LR to give relative toxicity. When toxicity factors were reported in multiple metrics and differed among references, we averaged the reported values. Only studies that included MC-LR and at least one other MC congener were considered. The toxicities of congeners besides MC-LR, MC-RR, and MC-YR have been under-studied, and their relative toxicity might be based on just one study (Table 1). For congeners without toxicities reported in the literature, we used relative toxicity of 1.0 to be similar to MC-LR.

For the experiments, again, all 12 MC congeners were summed to calculate the total MC concentration. Because MC-RR, MC-LR, MC-YR, and MC-LA were the four most common and highest concentration congeners detected, the remaining MC congeners were pooled and summed. Then, the percentage of MC-RR, MC-LR, MC-YR, MC-LA, and the other MCs of the total MCs were calculated. A multivariate analysis of variance (MANOVA) was conducted in SPSS (version 27) on each experiment to determine if incubation and nutrient enrichments affected the congener profile. When significant (*p* < 0.05, using the Wilks’ Lambda value), an ANOVA with a post-hoc Tukey test was conducted on each congener to determine differences among the treatments. Then, the treatments were ranked from highest to lowest and assigned a value from 5 (the treatment with the highest %MC-XX) to 1 (the lowest percentage). Then, across all experiments and within each congener, the treatment ranks were averaged and analyzed by ANOVA with a post hoc Tukey test to determine differences among treatments.

## 3. Results

### 3.1. Lake survey data

Cyanobacteria-specific chl *a* concentrations were less than 5 μg/L at all sites during June and early July of both years (Supplemental Fig. 1). Cyanobacteria-specific chl *a* concentrations peaked in late July to early August of both years, and 2019 had ~5 times greater concentrations than 2018. The Maumee Bay sites had the highest cyanobacteria-specific chl *a* concentrations, and concentrations decreased with increasing distance from Maumee Bay. The Islands and Central Basin sites had similar cyanobacteria-specific chl *a* concentrations between the two years. Cyanobacteria-specific chl *a* concentrations decline throughout September to low concentrations (< 5 μg/L) by the end of September.

*Microcystis* dominated the cyanobacteria community by accounting for between 50% to greater than 90% of all cyanobacteria biovolume at both sites and both years during July, August, and September (Supplemental Fig. 2), as seen in previous years. *Microcystis’s* dominance is particularly evident during the peak of the large bloom (2019) when it accounted for 92% to 98% of cyanobacteria biovolume. *Merismopedia* was sub-dominate to *Microcystis* at MB18 during 2018. *Planktothrix* was a minor (< 10% of total cyanobacteria biovolume), expect for MB18 in October 2019. *Dolichospermum* was a minor (< 10% of total cyanobacteria biovolume), expect for at WB83 on July 9 2019,. *Aphanizomenon* and *Chroococcus* were also observed at low levels in 2018.

Nutrient concentrations measured during 2018 and 2019 followed usual spatial and temporal pattern observed in Lake Erie (Supplemental Fig. 3–7). Nitrate concentrations peaked in early July (of both years) in Maumee Bay (> 300 μmol/L) and decreased to low, nitrogen-limiting concentrations (< 8 μmol/L) throughout August and September and decreased with increasing distance from Maumee Bay. Ammonium concentrations were much lower than nitrate (< 20 μmol/L), but ammonium concentrations were greatest in Maumee Bay during 2019. Total N showed a similar spatial and temporal pattern as nitrate. Dissolved reactive phosphorus concentrations were greatest in Maumee Bay during June and early July of both years (> 2 μmol/L), and DRP concentrations decreased from Maumee Bay and throughout August and September. Total P concentrations were relatively stable temporally in both years, and Maumee Bay had the highest concentrations (~2 to 8 μmol/L, or ~62 to 248 μg P/L).

**Fig. 2.**
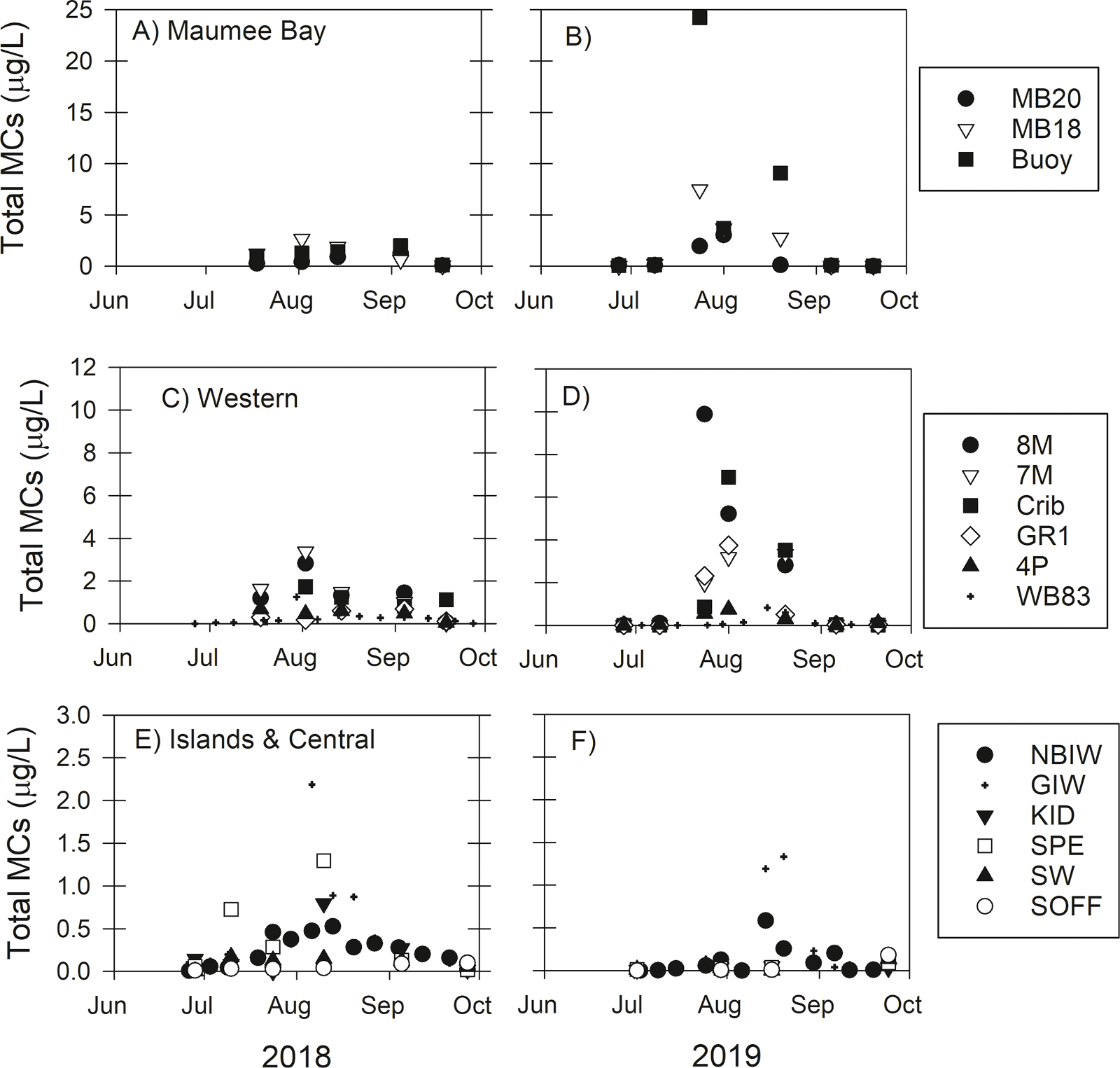
Total microcystin concentration at 15 sites in Lake Erie. The panels show data for sites in Maumee Bay (A, B), western Lake Erie (C, D), and around the islands on the border of the western and central basin (E, F), during the years of 2018 (left column) and 2019 (right column). Total microcystins is the sum of 12 congeners measured by LC-MS/MS. Note the different scales of the Y-axes of the panels.

Total MCs concentrations (the sum of 12 congeners) followed similar spatial and temporal patterns between 2018 and 2019, but peak total MCs were nearly an order of magnitude greater during 2019 (Fig. 2). In both years and across all sites, total MCs were in low concentration (< 1 μg/L) in late June and early July, increased throughout July and peaked in late July through early August. Total MCs during peak concentrations in 2019 were in highest concentrations at the Maumee Bay sites (MB20, MB18, and buoy; 1.95 μg/L to 24.24 μg/L) and were the lowest (< 1.3 μg/L) at the sites around the Islands and Central Basin (Fig. 2). Total MCs in September 2018 (concentrations up to 2.0 μg/L) were greater in September 2019 (all samples less than 0.04 μg/L). Total MCs were highly correlated with cyanobacteria-specific chl *a* concentrations (*r* = 0.759, *P* < 0.001; Supplemental Table 12). Total MCs were also positively correlated with the concentration of TN (*r* = 0.327) and TP (*r* = 0.379). However, due to the time delay between high nutrient concentrations (in June) and peak cyanobacterial bloom (late July and early August), very low MCs were also observed in high nutrient concentration waters (Supplemental Fig. 8).

All 12 MC congeners were detected in both years, but the congeners differed in frequency of detection and concentration (Table 2). Across both years and all sites, MC-LR was the most commonly detected congener (detected in 91.0% of all 223 samples), followed by MC-LA (78.5%), MC-RR (75.3%), and MC-YR (57.0%), which were the only congeners detected in more than 50% of the samples. These four congeners also had the four highest maximum and average concentrations recorded with MC-RR and MC-LR detected in the highest and second-highest concentrations, respectively.

**Fig. 3.**
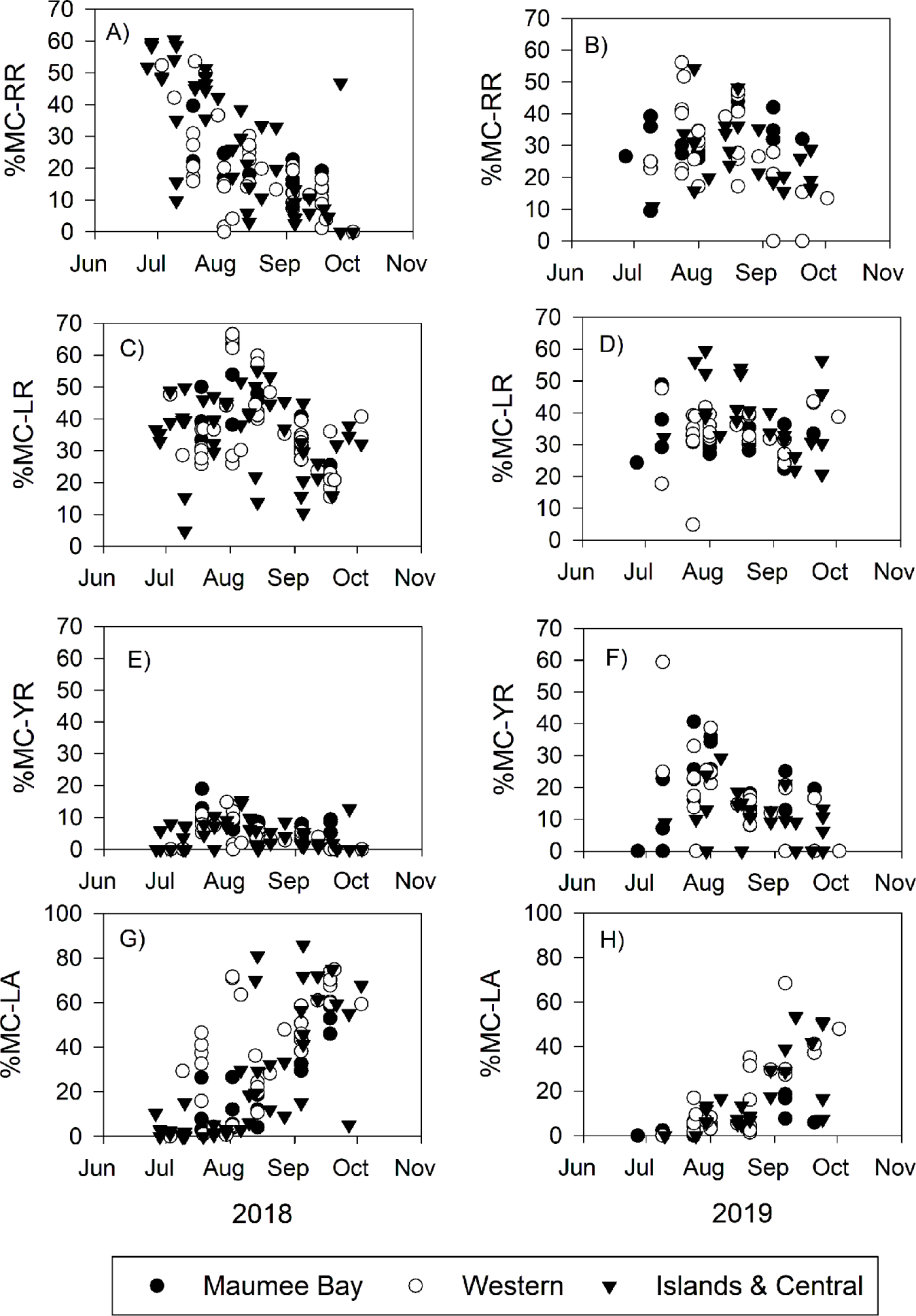
Microcystin congeners in the western basin of Lake Erie as the percent of total microcystins as a function of date during the years of 2018 (left column) and 2019 (right column). The panels are ordered from the congener with the highest%N (MC-RR) to the lowest%N (MC-LA).

**Fig. 4.**
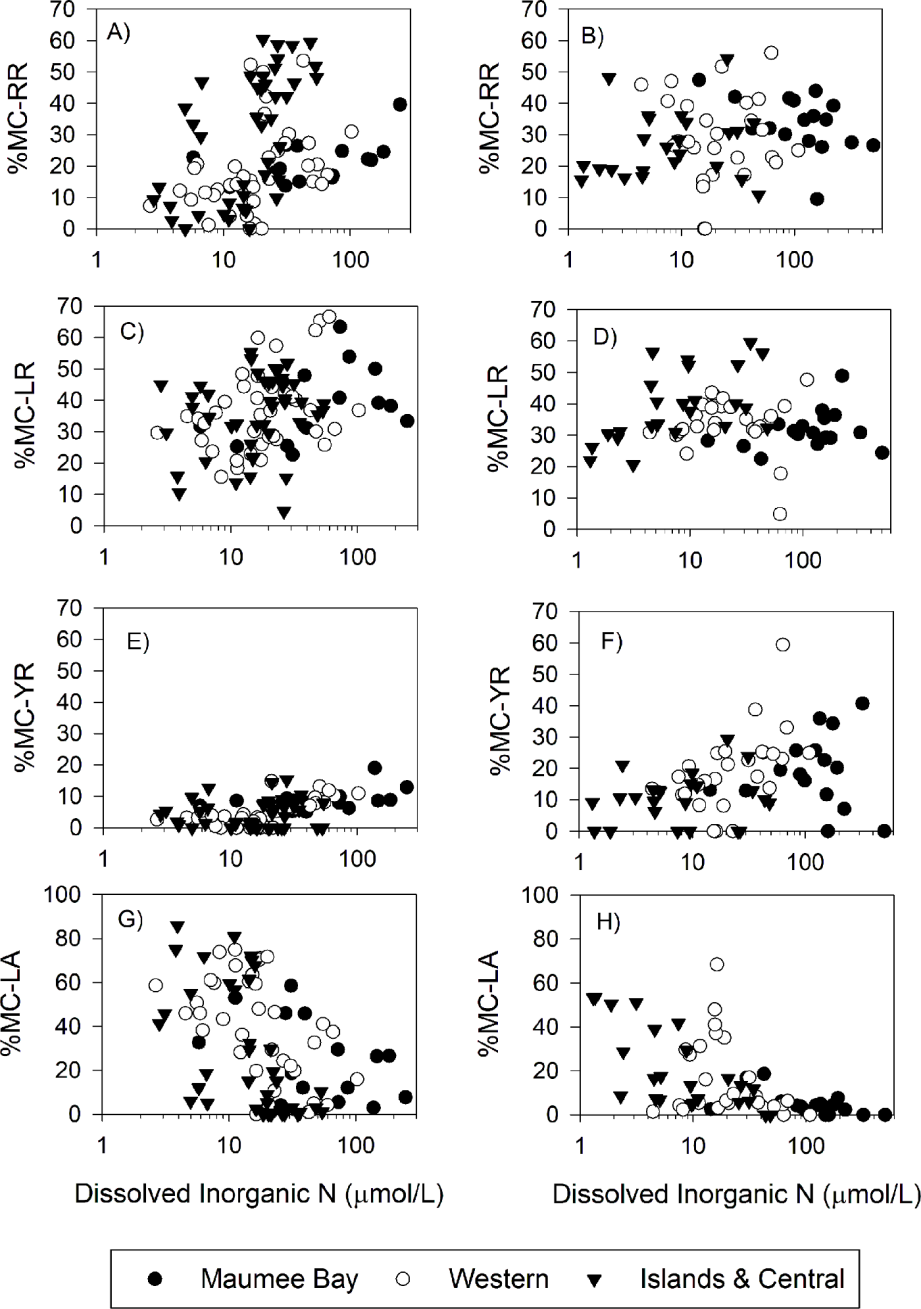
Microcystin congeners in the western basin of Lake Erie as the percent of total microcystins as a function of dissolved inorganic nitrogen concentration in the years of 2018 (left column) and 2019 (right column). The panels are ordered from the congener with the highest %N (MC-RR) to the lowest %N (MC-LA).

The MC congener profile was significantly affected by the day of year (*P* < 0.001), year (*P* < 0.001), site grouping (*P* < 0.001), and the interaction between year and site (*P* = 0.007, Supplemental Table 1). Plotting the percent congener (abbreviated as %MC-XX) against time showed that the MC congener profile changed seasonally (Figs. 3 and 4). In both years, %MC-LR ranged from 20% to 50% in the majority of the samples and did not have a significant temporal or spatial pattern (Fig. 3; (Supplemental Table 1). During 2018, %MC-RR was 40% to 60% in July and decreased to less than 20% by the end of September, while during 2019, %MC-RR was variable during July, ranging from 11.2% to 56.1% and declined to less than 30% by the end of September. The MANCOVA Partial Eta Squared (η^2^) indicated that day of year explained 26.2% of the variability of%MC-RR (Supplemental Table 1). During 2018, the majority of the samples high in %MC-RR were from the locations in the near Islands and Central Basin sites, but this difference was not significant (*P* = 0.169) and had a η^2^ of only 8.4%, and this spatial pattern was not observed in 2019 (Fig. 3). In both years, %MC-LA increased from less than 10% during July to greater than 60% in September (Fig. 3), and the η^2^ for day of year was 47.7% for %MC-LA (Supplemental Table 1). %MC-YR differed between the years 2018 and 2019 (η^2^ was 26.7%, Supplemental Table 1). During 2018, %MC-YR was less than 15% all year, but during 2019, %MC-YR ranged from 10% to 40% in July and August 2019 and then decreased in September to less than 20% (Fig. 3).

The seasonal changes in %MC-RR were positively correlated with dissolved inorganic nitrogen concentration (the sum of nitrate, nitrite, and ammonium, DIN) (*r* = 0.218, *P* < 0.01; supplemental Table 2), whereas %MC-LA was negatively correlated with DIN (*r* = −0.467, *P* < 0.01). These temporal patterns in %MC-RR and %MC-LA and DIN concentration were observed across all three site groups. The correlations of MC-RR and MC-LA with DIN were stronger in 2018 than in 2019. Similar but more variable correlation patterns were observed with these congeners at total N concentration (supplemental Fig. 9; supplemental Table 2) and the ratio of total N to total P (supplemental Fig. 10; supplemental Table 2). There were no apparent correlations between these congeners and total P concentration (supplemental Fig. 11; supplemental Table 2). MC-YR had a similar correlation pattern as MC-RR. MC-LR did not correlate with any environmental variable (*P* > 0.05 or *r* < 0.2 when the P value was < 0.05). Three of the less common MCs (MC–HtyR, MC–HilR, and MC-WR) were positively correlated with cyanobacteria chl *a* and total MC concentrations (*r* > 0.55; P < 0.01; supplemental Table 2)

**Fig. 5.**
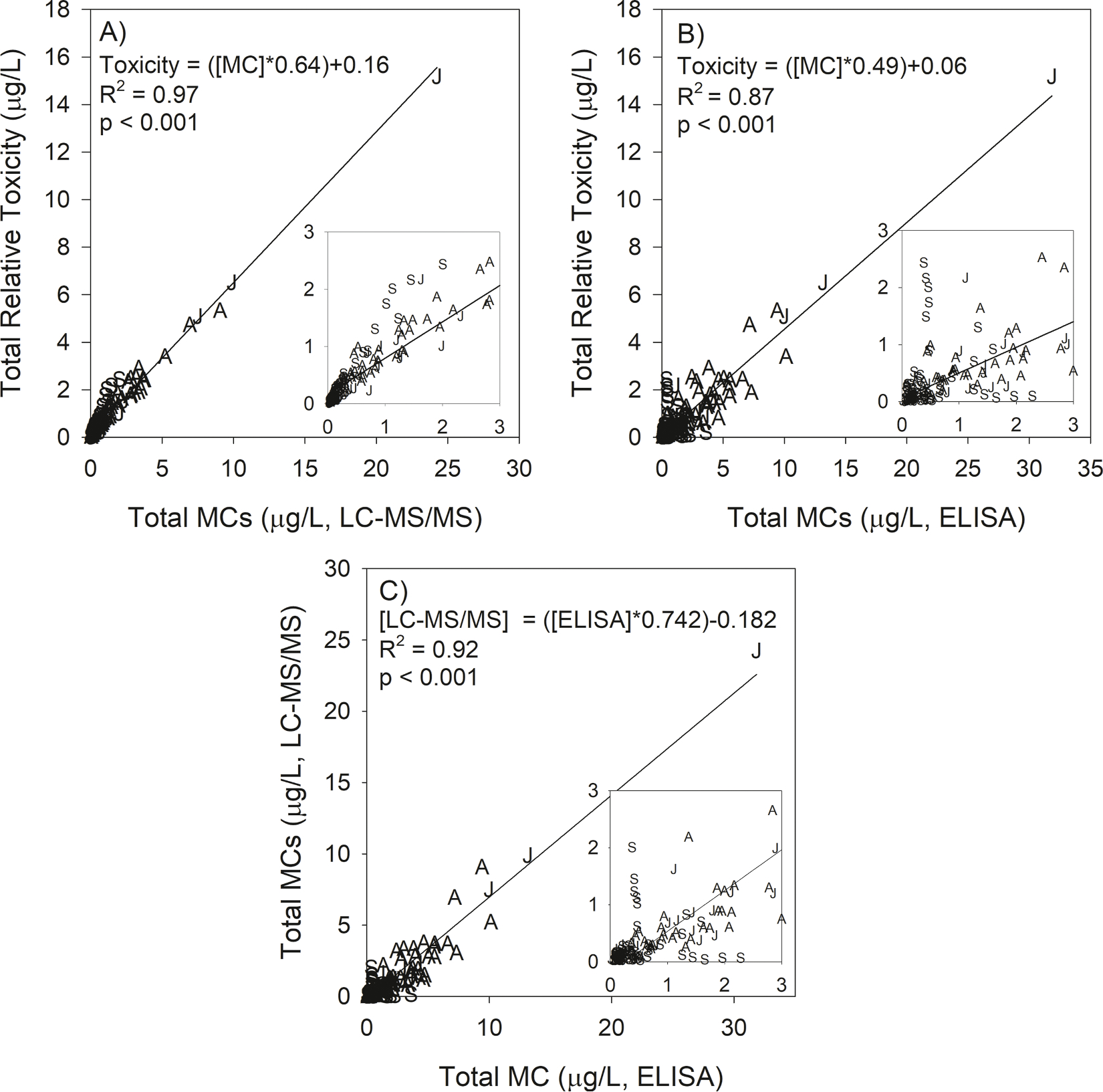
Relative toxicity vs total MC concentration as measured by LC-MS/MS (A) and ELISA (B) and the relationship between ELISA and LC-MS/MS (C). Letters on the graph tell which month the sample was collected (*J* = July, *A* = August, *S* = September). Total relative toxicity is the sum of individual congener concentrations multiplied by its relative toxicity (Table 1). The subfigure is the same data zoomed into a lower range to show more September samples falling above the trend line.

Total relative toxicity increased linearly with total MC concentration by LC-MS/MS (R^2^_=_ 0.97, Fig. 5A). The slope of the regression line was 0.64, suggesting that using total MC concentration overestimates the toxicity by one-third. Total relative toxicity increased with ELISA-measured total MC-LR equivalents, and the relationship had more variability (as indicated by a lower R^2^_=_ 0.87; Fig. 5B). The regression line slope was 0.49, which suggests that the relative toxicity of a sample is less than half as estimated by ELISA. There was a significant linear relationship between the two methods (R^2^_=_ 0.92, Fig. 5C), but there was much variability at ELISA concentrations less than 3.0 μg/L. The regression line slope was 0.74, which suggests ELISA generates a higher concentration than LC-MS/MS.

**Fig. 6.**
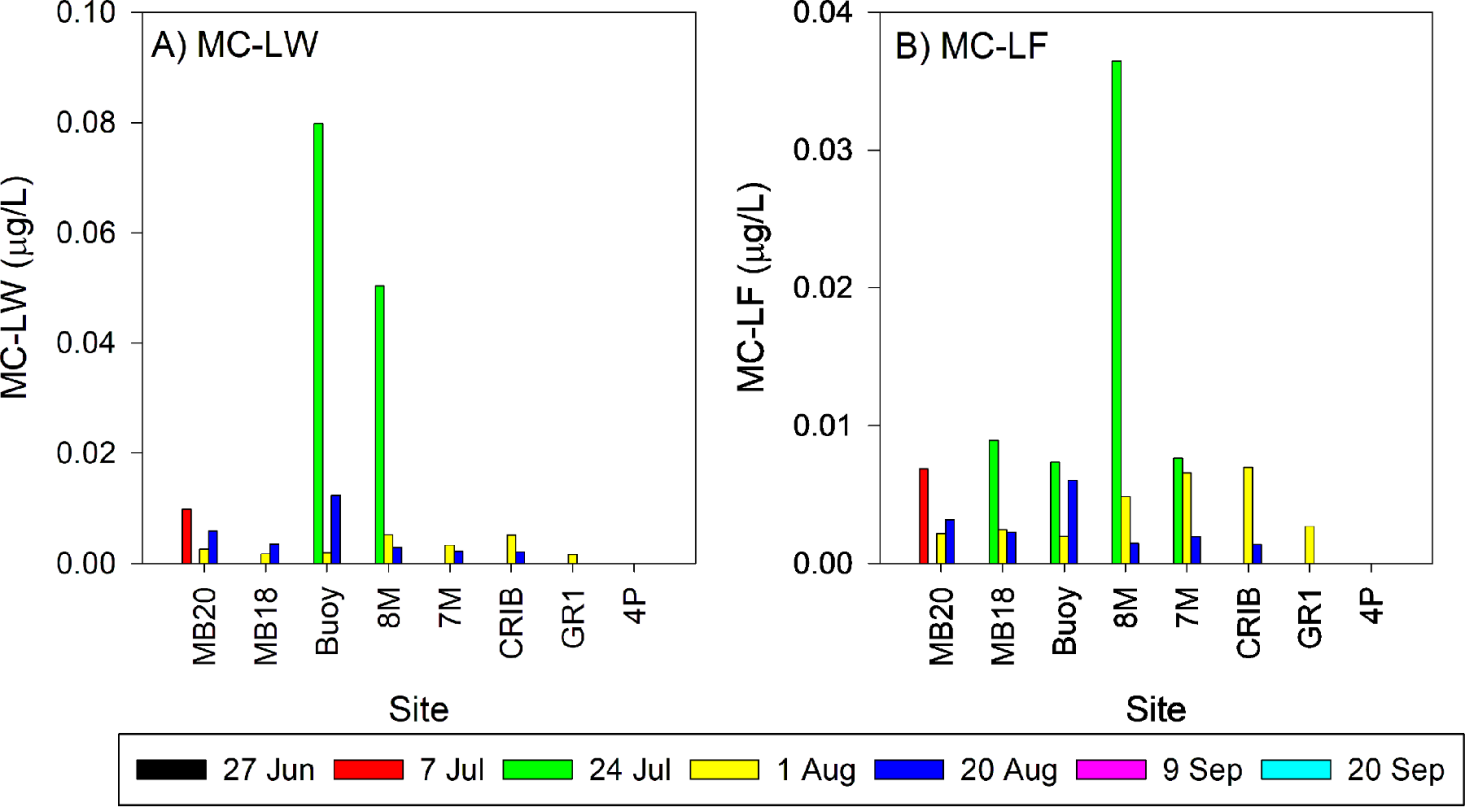
Concentrations of the highly toxic MC-LW and MC-LF measured during 2019 in Maumee Bay and western Lake Erie. The sample sites on the X axis are arranged by increasing distance from the Maumee River.

The two most-toxic congeners, MC-LW and MC-LF, have a relative toxicity of 7.0 (Table 1). MC-LW and MC-LF were only detected in 1.8% and 3.6%, respectively, of all samples collected during 2018 (Table 2). MC-LW and MC-LF were detected at higher frequencies during 2019 at 17.1% and 18.0% samples, respectively. Sites Buoy and 8 M on July 24, 2019, had relatively high MC-LW (0.080 and 0.051 μg/L, respectively) and MC-LF (0.007 and 0.037 μg/L, respectively) concentrations (Fig. 6). Multiplying each congener concentration by the relative toxicity factor of 7 and summing those values gives the relative toxicity (associated with just MC-LW and MC-LF) of 0.61 and 0.62 μg/L for Buoy and 8 M, respectively. 84% and 90% of the MC-LW and MC-LF detections occurred in Maumee Bay and the western sites, and the highest concentrations of MC-LW and MC-LF occurred in waters with TP and TN concentrations exceeding 1.5 μmol/L and 70 μmol/L, respectively (Supplemental Fig. 14).

### 3.2. Experimental results

Total MCs concentrations in the post-incubation samples were higher or similar to the initial concentrations, indicating that total MCs did not decrease throughout any experiment (supplemental Figs. 15 and 16). Twelve of the 18 experiments had significant differences among initial and post-incubation treatments. In general, adding P and N resulted in higher total MCs than the control (supplemental Figs. 15 and 16). Although differences in total MC among the N treatments (nitrate, ammonium, urea) were found in a few experiments, no N form consistently resulted in a higher or lower total MC than the other N forms.

**Fig. 7.**
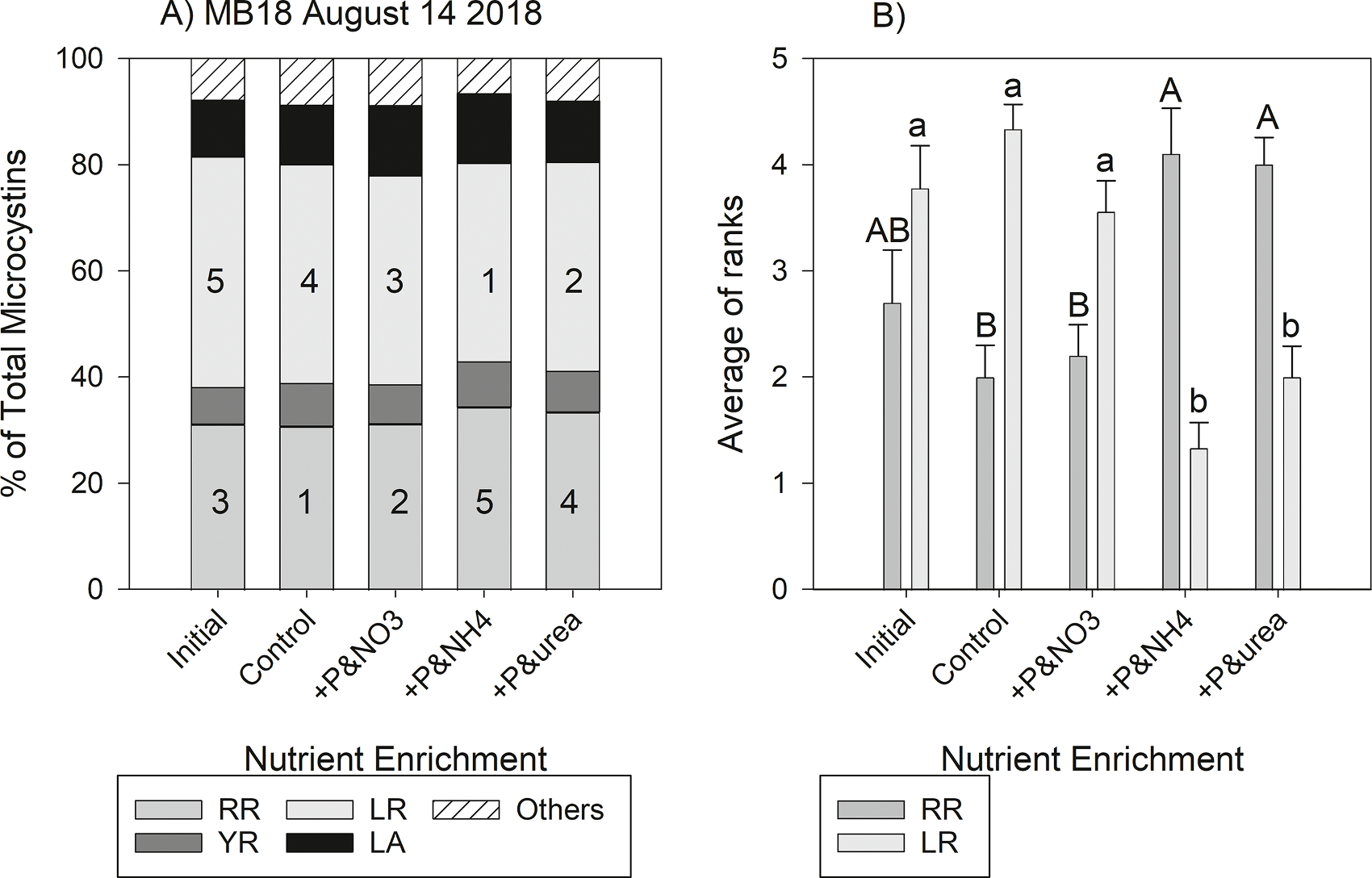
A) Microcystin congeners as the percent of total microcystins in the nutrient enrichment experiment at site MB18 that began on August 14, 2018. MC-RR% and MC-LR% significantly differed among treatments, and the ranks of the percent congeners in the treatments are superimposed onto the graph (i.e., 5 > 4 > 3 > 2 >1). MC-YR and MC-LA did not have significant differences among treatments in this experiment. B) The average rank (± standard error) of MC-RR% and MC-LR% among the treatments show that the phosphate and ammonium (+*P*&NH4) and phosphate and urea (+*P*&urea) treatments resulted in significantly higher MC-RR% (*p* < 0.001, upper case letters) and significantly lower MC-LR% (*p* < 0.001, lower case letters) than the other treatments. The letters above the bar are post-hoc Tukey test groupings (the mean of *A* > mean of B).

The four most common MC congeners (MC-LR, -RR, -YR, and -LA) observed were also at the highest concentrations in the experiments. The MANOVA suggested significant differences among the MC congener profile in 12 of the 18 experiments (Table 3). Among these 12 experiments, %MC-RR differed among initial, control, and the three P and N enrichment treatments in 10 experiments, %MC-YR in 6 experiments, % MC-LR in 9 experiments, and %MC-LA in 4 experiments. While there were significant differences, the absolute differences among treatments were relatively small, which may be attributed to the short incubation time of 3 days. For example, in the Maumee Bay experiment that began on August 14, 2018, %MC-RR was 31.11% (±1.09% standard error) in the initial samples and increased to 34.36% (± 0.60%) after 72 h in the P & ammonium enrichment (Fig. 7A). In this same experiment, %MC-LR was 43.45% (± 1.51%) in the initial sample and it decreased to 37.43% (± 1.33%) after 72 h in the P & ammonium enrichment. In this experiment, %MC-RR and %MC-LR were significantly different among treatments (despite the modest change in overall percentage), but %MC-YR and %MC-LA were not different among treatments (see August 14, 2018, in Table 3).

For the congener ranks analysis, in each experiment a value of 5 was assigned to the treatment with the highest percentage (e.g., a value of 5 was assigned to the P & ammonium treatment for%MC-RR in the experiment in Maumee Bay on August 14, 2018), whereas a value of 1 was assigned to the treatment with the lowest percentage (the control received a 1 for %MC-RR in this experiment; Fig. 7A). Across all experiments with significant differences indicated by the MANOVA, the P & ammonium and P & urea resulted in the highest rank for %MC-RR (4.1 ± 0.43 and 4.0 ± 0.26 respectively), indicating that these treatments resulted in higher %MC-RR, whereas the control (2.0 ± 0.24) resulted in the lowest %MC-RR (Fig. 7B). The control had the highest MC-LR (sum of rank was 4.3 ± 0.24), whereas P & ammonium and P & urea resulted in the lowest %MC-LR (1.3 ± 0.24 and 2.0 ± 0.29, respectively). The MC congener profile and assigned ranks for every experiment can be seen in the supplemental document (supplemental Figs. 17 and 18).

## 4. Discussion

The four most common and highest concentration MC congeners reported in this study, MC-LR, MC-RR, MC-YR, and MC-LA, agree with a study by Palagama et al. (2020). Both studies used the same sampling sites, reported *Microcystis* as the dominant cyanobacteria, and were performed in consecutive years (2016–2017 for Palagama et al. (2020), and 2018–2019 for the present study). Although Palagama et al. (2020) identified 27 MC congeners, only MC-LR, MC-RR, MC-YR, and MC-LA were above quantifiable levels. Since the concentration of the other MC congeners were very low compared to the four main congeners (in both studies), it is unlikely the total MCs reported here would have significantly differed if all 27 MC congeners analyzed by Palagama et al. (2020) were included in this study. However, other MC congeners have been found in relatively higher concentrations in other areas of the Lake Erie system. Palagama et al. (2020) reported MC-LF and MC-LW in a Maumee River *Microcystis* September 2017 bloom that accounted for up to 15.2% and 17.4% of the total MCs. Globally, while MC-LR and MC-RR are usually the most frequently encountered, there have been numerous reports of MCs other than the four common congeners found in Lake Erie accounting for a substantial percentage of the total MCs, for example, MC-FR, MC-WR, MC-LW, and MC-LF, as reviewed by Díez-Quijada et al. (2019). Information on what drives the production of each congener is scarce and needed, given that some rarer congeners are much more toxic than MC-LR (Table 1).

The shift in dominant congeners from MC-LR and MC-RR to MC-LR and MC-LA observed in Lake Erie occurred gradually throughout two to three months (Fig. 3), and this shift corresponded to a drawdown of dissolved inorganic N concentration (Fig. 4). Of the four main MC congeners in Lake Erie, MC-RR has the highest N demand (Table 1). Likewise, experimental enrichments of reduced N forms (ammonium and urea), which are cyanobacteria’s preferred N source because they require lower energy for incorporation than nitrate (Flores and Herrero, 2005), resulted in significantly more MC-RR in 9 of the 18 experiments (Table 3). Hence, when N was available in excess in the lake and in our microcosm experiments, *Microcystis* produced the MC congener with the highest N demand. On the other hand, MC-LA has the lowest N demand, and increased in percentage as N concentrations declined in the lake. Collectively, our findings build upon previous studies that showed N availability plays a major role in total MC production (Ginn et al., 2010; Harke and Gobler, 2015; Horst et al., 2014) and that the MC congener profile changes with N availability in accordance with the expectations of ecological stoichiometry (Van de Waal et al., 2014, 2009; Wagner et al., 2019).

The gradual transition in the MC congener profile observed in the lake agrees with the small but often significant changes in the congener profile in the short-term experiments. After three days of incubation with extra N, MC-RR increased only by 3.26% in the exemplar experiment shown in Fig. 7A. Although *mcy* gene expression can double within hours of a N pulse (Chaffin et al., 2018; Harke and Gobler, 2015), the congeners were produced in a similar percentage as before the N addition. Likewise, a culture experiment with *Microcystis* strain CAWBG11 showed that MC-RR slowly decreased throughout a 36-day incubation while nitrate was depleted (Puddick et al., 2016). These observations suggest that the strains present will utilize their current cellular machinery to synthesize MCs following an environmental stimulus, but changes in the MC congener profile at the cellular level of individual strains and at the ecosystem-scale take weeks to months, not days.

Different strains of *Microcystis* and variations within the *mcy* operon can affect congeners (Mikalsen et al., 2003). For example, within the *mcyB* gene, the *mcyB* C1-like genotype produces both MC-LR and MC-RR, whereas the *mcyB* B1-like genotype only produced MC-LR (Yancey et al., 2022). The C1 genotype is found in higher numbers than the B1 genotypes in Lake Erie (Yancey et al., 2022), which aligns with our observation of high%MC-RR (Fig. 3). *Microcystis* strain LE19–195.1 from the Western Lake Erie Culture Collection produced equal amounts of MC-YR as MC-LR in culture, and this strain was collected from western Lake Erie during early August 2019 (Yancey et al., in press) when %MC-YR was as high as 40% (Fig. 3F). The presence of LE19–195.1 in Lake Erie during 2019 might explain the difference between %MC-YR in 2018 (< 15% all year) and in 2019 (up to 60%). The MC congener profile is likely a function of the interaction among environmental factors, how these factors select for different MC-producing strains and species, and how they affect the MC production rate among the toxic strains. The rapid advancements of genomic technologies will soon provide a greater understanding of MC-producing strains in lakes.

The MC congeners have a wide range of toxicity (Table 1). The early bloom phases (July) were characterized by higher total MC concentrations dominated by MC-LR and the less toxic MC-RR, whereas the late bloom phases (September) were characterized by lower total MC concentrations but dominated by MC-LR and the more toxic MC-LA (Figs. 2 and 3). The shift towards more-toxic congeners did not result in higher toxicity because the decrease in total MC concentration more than compensated for an increased percentage of highly toxic congeners. (Fig. 5). Moreover, the slope of total MCs vs. total toxicity was 0.64, indicating that in general, reports of total MCs by LC-MC/MS overestimate toxicity by 56%. Furthermore, the ELISA method overestimated toxicity by 104%. In other words, it would take a typical sample from Lake Erie to have an ELISA value of 2.04 μg/L to reach the same toxicity as the current World Health Organization lifetime guideline for drinking water of 1 μg/L of MC-LR. However, several samples collected in September had a total relative toxicity value that was greater than the LC-MS/MS-measured total MC concentration measured (Fig. 5A insert). These samples had measured total MC concentrations ranging from 0.5 to 2 μg/L, but the total relative toxicity ranged from 1.0 to 2.5 μg/L. In these samples, the measured total MCs underestimated toxicity.

The two most-toxic MC congeners detected were MC-LW and MC-LF, which have a relative toxicity of 7.0 (Fischer et al., 2010). MC-LW and MC-LF were more frequently found in 2019 than in 2018 (Table 2), and these congeners were mainly found in the nutrient-rich waters of Maumee Bay and the locations closest to shore (Fig. 6, supplemental Fig. 6). Additionally, during the 2017 *Microcystis* bloom in Maumee River, Palagama et al. (2020) reported MC-LF and MC-LW concentrations as high as 20.21 μg/L and 25.54 μg/L, respectively. These findings may suggest that eutrophic conditions promote these toxins. While the maximum concentrations of MC-LW (maximum concentration of 0.080 μg/L) and MC-LF (maximum of 0.036 μg/L) in our study were low compared to other MC congeners, their contribution to relative toxicity (up to 0.62 μg/L) alone could have been high enough to exceed Ohio’s drinking water threshold for young children (0.3 μg/L) if not adequately removed by water treatment. The high concentrations of MC-LW and MC-LF suggest that routine monitoring of the MC congener profile is needed near river mouths and nearshore zones where many drinking water intakes and recreational areas are located.

Hellweger and coworkers (Hellweger et al., 2022) created a MC production model based on a broad literature meta-analysis that suggested that the 40% P load reduction planned for Lake Erie (GLWQA, 2015) without a concurrent N load reduction would make the cyanobacterial bloom more toxic. Hellweger et al. (2022) suggest that the lower P concentration would reduce overall phytoplankton and cyanobacterial biomass, allowing dissolved inorganic N to persist longer into the growing season and the water to be clearer. The available N and clear water would select for MC-producing strains and result in higher MC production rates. However, their model does not discriminate between the MC congeners. Our data suggest that the MC congener profile also depends on N availability, and the high concentration of N selects for MC-RR, a less toxic MC. Hence, our results indicate that P load reduction of Lake Erie without concurrent N load reduction is likely to favor less toxic MC congeners. A recent rebuttal to Hellweger et al. (2022) also pointed this out (Huisman et al., 2022). On the other hand, because MC-LR did not correlate with DIN (Fig. 4) and nitrate enrichment favored MC-LR over MC-RR (Fig. 7B), it remains unknown how MC-LR will be affected by a P-only load reduction. In other words, would MC-LR be unchanged in concentration while decreasing in percentage of total MCs (increasing toxicity, as predicted by Hellweger et al. (2022)), or would the MC congener profile shift to predominantly MC-RR (decreasing toxicity, as argued by Huisman et al. (2022)).

In conclusion, the MC congener profile in Lake Erie shifted from domination by MC-LR and MC-RR to MC-LR and MC-LA over a span of two to three months, and this shift corresponded to a drawdown of dissolved inorganic N concentration. Conversely, experimental results showed that enrichments of reduced N stimulated MC-RR production. Collectively, these results agree with previous ecological stoichiometry studies (Van de Waal et al., 2009) suggesting that N-rich waters favor the production of N-rich MC congeners (such as MC-RR), while N-limiting waters favor lower N MC congeners (such as MC-LA). Furthermore, during early summer (July), when total MC concentrations may be high, overall MC toxicity in Lake Erie tends to be significantly overestimated due to the presence of less-toxic congeners such as MC-RR. On the other hand, in the fall (September), the total MC concentrations are low, but the presence of more-toxic congeners such as MC-LA may result in an underestimation of overall toxicity. While present only at low concentrations, the most-toxic congeners, MC-LW and MC-LF, tend to be common near the mouth of the Maumee River, which suggests that nearby drinking water intakes and beaches should be examined more closely. Additionally, these more-toxic MCs are hydrophobic (Vesterkvist and Meriluoto, 2003) and more prone to aerosolization than the less-toxic, hydrophilic MC-RR (Olson et al., 2020). We recommend continued monitoring of the MC congener profile, in addition to the measurements of total MCs, to better understand the toxicity of cyanobacterial blooms in Lake Erie.

## Supplementary Material

supplementary material

## Figures and Tables

**Table 1:** The 12 microcystin congeners tested for in the western basin during 2018 and 2019, their percent nitrogen by mass, the carbon: nitrogen ratio (by atoms), the method limit of quantification (MLQ) in parts per trillion (ppt, ng/L), and toxicity relative to MC-LR. When no toxicity reference was found, 1.0 was used for relative toxicity. When multiple citations reported differences in toxicity, we averaged the values. References – 1: Chernoff et al., 2021; 2: Faassen and Lürling 2013; 3: Gupta et al., 2003; 4 Fischer et al. 2010.

Congener	Chemical	Percent N by mass	C:N (by atoms)	MLQ (ppt)	Relative Toxicity	Reference

[D-Asp^3^]MC-RR	C_47_H_72_N_13_O_12_	18.01	3.62	5	1.00	No reference, used 1.0
MC-RR	C_49_H_76_N_13_O_12_	17.52	3.77	5	0.21	Average of 1, 2, 3
MC-YR	C_52_H_73_N_10_O_13_	13.39	5.2	5	0.50	Average of 1, 2, 3
MC—HtyR	C_53_H_74_N_10_O_13_	13.22	5.3	10	0.50	Similar to MC-YR (1)
MC-LR	C_49_H_75_N_10_O_12_	14.06	4.9	1	1.00	Basis
[D-Asp^3^]MC-LR	C_49_H_73_N_10_O_13_	13.87	4.9	1	1.00	No reference, used 1.0
MC—HilR	C_50_H_76_N_10_O_12_	13.88	5.0	5	1.00	No reference, used 1.0
MC-WR	C_54_H_74_N_11_O_12_	14.41	4.91	5	1.00	No reference, used 1.0
MC-LA	C_46_H_68_N_7_O_12_	10.76	6.57	5	2.34	1
MC-LY	C_52_H_72_N_7_O_13_	9.77	7.43	5	0.78	Average of 1 and 2
MC-LW	C_54_H_73_N_8_O_12_	10.92	6.75	5	7.00	4
MC-LF	C_52_H_72_N_7_O_12_	9.93	7.43	5	7.00	4

**Table 2:** Summary of quantified microcystin congeners in the western basin during 2018 and 2019.

	Both years (*n* = 223)			2018 (*n* = 112)			2019 (*n* = 111)		
	% detections	Max (μg/L)	Average (μg/L)	% detections	Max (μg/L)	Average (μg/L)	% detections	Max (μg/L)	Average (μg/L)

D-Asp^3^ RR	19.3	0.526	0.033	11.6	0.526	0.054	27.0	0.147	0.021
MC-RR	75.3	9.900	0.310	82.1	0.571	0.125	68.5	9.900	0.547
MC-YR	57.0	3.900	0.188	59.8	0.444	0.057	54.1	3.900	0.357
MC—HtyR	24.2	0.516	0.037	25.9	0.039	0.012	22.5	0.516	0.079
MC-LR	91.0	7.964	0.294	98.2	2.205	0.221	83.8	7.964	0.381
D-Asp^3^ LR	47.5	0.168	0.010	58.0	0.039	0.008	36.9	0.168	0.014
MC—HilR	31.8	0.303	0.033	35.7	0.044	0.013	27.9	0.303	0.060
MC-WR	17.5	0.497	0.038	15.2	0.021	0.010	19.8	0.497	0.061
MC-LA	78.5	0.672	0.098	92.0	0.672	0.122	64.9	0.461	0.063
MC-LY	25.6	0.294	0.018	23.2	0.012	0.005	27.9	0.294	0.030
MC-LW	9.4	0.080	0.009	1.8	0.005	0.002	17.1	0.080	0.010
MC-LF	10.8	0.036	0.005	3.6	0.007	0.004	18.0	0.036	0.006

**Table 3:** MANOVA and Tukey test summary table of the four most common microcystin congeners in the nutrient enrichment experiments. NS = not significant Tukey test. *I* = initial values. C = control, *N* = phosphate and nitrate, *A* = phosphate and ammonium, *U* = phosphate and urea. Treatments listed under each congener are ordered lowest average to highest.

			Wilk’s Lambda		Tukey test	Tukey test	Tukey test	Tukey test
Year	Date	Site	MANOVA f value	p value	MC-RR	MC-YR	MC-LR	MC-LA

2018	2 August	MB18	0.928	0.553	NS	NS	NS	NS
	14 August	MB18	2.603	0.019	C N I U A	NS	A U N C I	NS
	4 September	MB18	1.973	0.069	NS	NS	NS	NS
	18 September	MB18	0.574	0.871	NS	NS	NS	NS
	9 October	MB18	0.541	0.894	NS	NS	NS	NS
2018	3 July	WB83	8.068	<0.001	C N A I U	NS	U A I N C	NS
	30 July	WB83	5.898	<0.001	N C U I A	I C A U N	A U I N C	NS
	13 August	WB83	9.698	<0.001	C N I A U	C U A I N	A U N C I	I U N A C
	27 August	WB83	8.361	<0.001	NS	NS	NS	NS
	19 September	WB83	5.973	<0.001	N A U C I	C N U A I	NS	NS
2019	2 July	MB18	3.317	0.005	NS	N I A U C	NS	C U A N I
	16 July	MB18	4.888	<0.001	N C U I A	NS	A U N C I	I A U C N
	13 August	MB18	3.093	0.008	I C U N A	NS	A N C U I	U C N A I
	28 August	MB18	4.320	0.001	I C N U A	N C U A I	A U I N C	NS
2019	13 July	WB83	1.039	0.458	NS	NS	NS	NS
	30 July	WB83	2.176	0.046	NS	NS	U I A N C	NS
	14 August	WB83	3.376	0.004	I N C U A	A N C U I	A U I C N	NS
	26 August	WB83	3.324	0.005	I C N U A	NS	NS	NS

## Data Availability

Data will be made available on request.
