# Supplementary material for: Microcystin congeners in Lake Erie follow the seasonal pattern of nitrogen availability
Source: Harmful Algae. Author manuscript; Available in PMC 2024 Feb 15. (PMC10867787; doi:10.1016/j.hal.2023.102466)
Supplement: supplementary material [file NIHMS1961302-supplement-supplementary_material.docx]

Supplemental Figures for Microcystin congeners in Lake Erie follow the seasonal pattern of nitrogen availability

Justin D. Chaffin^a^*, Judy A. Westrick^b**^, Laura A. Reitz^c,1^, Thomas B. Bridgeman^d^

a: F.T. Stone Laboratory and Ohio Sea Grant, The Ohio State University, 878 Bayview Ave. P.O. Box 119, Put-In-Bay, OH 43456, USA

b: Lumigen Instrument Center, Wayne State University, 5101Cass Ave., Detroit, MI 48202, USA

c: Department of Biological Sciences, Bowling Green State University, Life Sciences Building, Bowling Green, OH 43402, USA

d: Lake Erie Center, University of Toledo, Oregon, OH, 43416, USA

1: Current address - Department of Earth and Environmental Sciences, University of Michigan, 2534 North University Building, 1100 North University Avenue, Ann Arbor, MI 48109-1005, USA

Supplemental Table 1. Multivariate analysis of covariance (MANCOVA) table of the percentage of MC-RR, MC-LR, MC-YR, and MC-LA of total microcystins in Lake Erie during 2018 and 2019. F and P values are from Pillai’s Trace statistic. Partial Eta Squared tells the proportion of total variation a predictor variable explains.

| MANCOVA Table |  |  |  |  |
| --- | --- | --- | --- | --- |
| Effect |  | F | P | Partial Eta Squared |
| Day of Year |  | 39.68 | <0.001 | 0.501 |
| Year |  | 22.11 | <0.001 | 0.359 |
| Site |  | 4.270 | <0.001 | 0.097 |
| Year * Site |  | 2.720 | 0.007 | 0.064 |
|  |  |  |  |  |
| ANCOVA Table |  |  |  |  |
| Effect | Dependent Variable | F | P | Partial Eta Squared |
| Day of Year | %MC-RR | 57.090 | <0.001 | 0.262 |
| Day of Year | %MC-LR | 10.870 | 0.001 | 0.063 |
| Day of Year | %MC-YR | 8.497 | 0.004 | 0.050 |
| Day of Year | %MC-LA | 147.200 | <0.001 | 0.477 |
| Year | %MC-RR | 7.686 | 0.006 | 0.046 |
| Year | %MC-LR | 2.216 | 0.139 | 0.014 |
| Year | %MC-YR | 58.680 | <0.001 | 0.267 |
| Year | %MC-LA | 32.740 | <0.001 | 0.169 |
| Site | %MC-RR | 1.796 | 0.169 | 0.084 |
| Site | %MC-LR | 1.064 | 0.348 | 0.206 |
| Site | %MC-YR | 7.660 | 0.001 | 0.103 |
| Site | %MC-LA | 4.517 | 0.012 | 0.121 |
| Year*site | %MC-RR | 0.896 | 0.410 | 0.011 |
| Year*site | %MC-LR | 2.966 | 0.006 | 0.062 |
| Year*site | %MC-YR | 5.310 | 0.054 | 0.036 |
| Year*site | %MC-LA | 0.905 | 0.407 | 0.011 |

Supplemental Table 2. Pearson correlation coefficient (r) matrix among environmental variables and microcystin (MC) congeners as the percent of total microcystins in Lake Erie during 2018 and 2019. Nutrient concentrations, cyanobacteria-chlorophyll, and total microcystins were log-transformed prior to correlation to obtain a normality distributed dataset. “NS” = not significant (P > 0.05). r values between with P values 0.05 and 0.01 are indicated by one asterisk (*) and P values less than 0.01 with two asterisks (**).

| Variable | NO_3+2_^-^ | NH_4_^+^ | DIN | TKN | TN | TN:TP | DRP | TP | Cyano-chl | Total MCs | ELISA MCs |
| --- | --- | --- | --- | --- | --- | --- | --- | --- | --- | --- | --- |
| Nitrate+Nitrite | 1 | .413^**^ | .991^**^ | .371^**^ | .805^**^ | .374^**^ | NS | .485^**^ | .199^*^ | NS | NS |
| Ammonium | .413^**^ | 1 | .495^**^ | .394^**^ | .528^**^ | -.298^**^ | .213^**^ | .641^**^ | .452^**^ | .317^**^ | .241^**^ |
| DIN | .991^**^ | .495^**^ | 1 | .412^**^ | .833^**^ | .326^**^ | .167^*^ | .539^**^ | .255^**^ | .184^*^ | NS |
| Total Kjdelhal N | .371^**^ | .394^**^ | .412^**^ | 1 | .789^**^ | -.162^*^ | .235^**^ | .796^**^ | .682^**^ | .524^**^ | .535^**^ |
| Total N | .805^**^ | .528^**^ | .833^**^ | .789^**^ | 1 | NS | .304^**^ | .800^**^ | .497^**^ | .327^**^ | .285^**^ |
| TN:TP | .374^**^ | -.298^**^ | .326^**^ | -.162^*^ | NS | 1 | NS | -.479^**^ | -.368^**^ | -.161^*^ | -.192^*^ |
| DRP | NS | .213^**^ | .167^*^ | .235^**^ | .304^**^ | NS | 1 | .296^**^ | NS | NS | NS |
| Total P | .485^**^ | .641^**^ | .539^**^ | .796^**^ | .800^**^ | -.479^**^ | .296^**^ | 1 | .656^**^ | .379^**^ | .360^**^ |
| Cyanobacteria-  chlorophyll *a* | .199^*^ | .452^**^ | .255^**^ | .682^**^ | .497^**^ | -.368^**^ | NS | .656^**^ | 1 | .759^**^ | .731^**^ |
| Total MCs | NS | .317^**^ | .184^*^ | .524^**^ | .327^**^ | -.161^*^ | NS | .379^**^ | .759^**^ | 1 | .806^**^ |
| ELISA MCs | NS | .241^**^ | NS | .535^**^ | .285^**^ | -.192^*^ | NS | .360^**^ | .731^**^ | .806^**^ | 1 |
| %MC-RR | .214^**^ | NS | .218^**^ | NS | .179^*^ | .208^**^ | NS | NS | NS | NS | NS |
| %MC-YR | .282^**^ | .353^**^ | .316^**^ | .308^**^ | .368^**^ | NS | .235^**^ | .280^**^ | .456^**^ | .336^**^ | .348^**^ |
| %MC-LR | NS | -.181^*^ | NS | NS | NS | .184^*^ | NS | NS | NS | NS | NS |
| %MC-LA | -.452^**^ | -.168^*^ | -.467^**^ | -.255^**^ | -.448^**^ | -.271^**^ | NS | -.230^**^ | -.318^**^ | -.264^**^ | -.251^**^ |
| %MC-LW | NS | NS | NS | NS | NS | NS | NS | .198^*^ | 0.096 | NS | NS |
| %MC-LF | NS | .182^*^ | NS | NS | .169^*^ | NS | NS | .217^**^ | NS | NS | NS |
| %MC-HtyR | NS | .154^*^ | NS | .426^**^ | .285^**^ | NS | NS | .324^**^ | .567^**^ | .689^**^ | .557^**^ |
| %Dasp^3^-MC-LR | NS | .158^*^ | NS | NS | .207^**^ | NS | NS | .204^**^ | .170^*^ | NS | NS |
| %MC-HilR | NS | .233^**^ | .166^*^ | .468^**^ | .315^**^ | NS | NS | .353^**^ | .607^**^ | .691^**^ | .597^**^ |
| %MC-WR | NS | .336^**^ | .162^*^ | .483^**^ | .318^**^ | -.253^**^ | NS | .433^**^ | .611^**^ | .664^**^ | .559^**^ |
| %MC-LY | NS | NS | NS | NS | NS | -.159^*^ | NS | NS | .208^**^ | NS | NS |


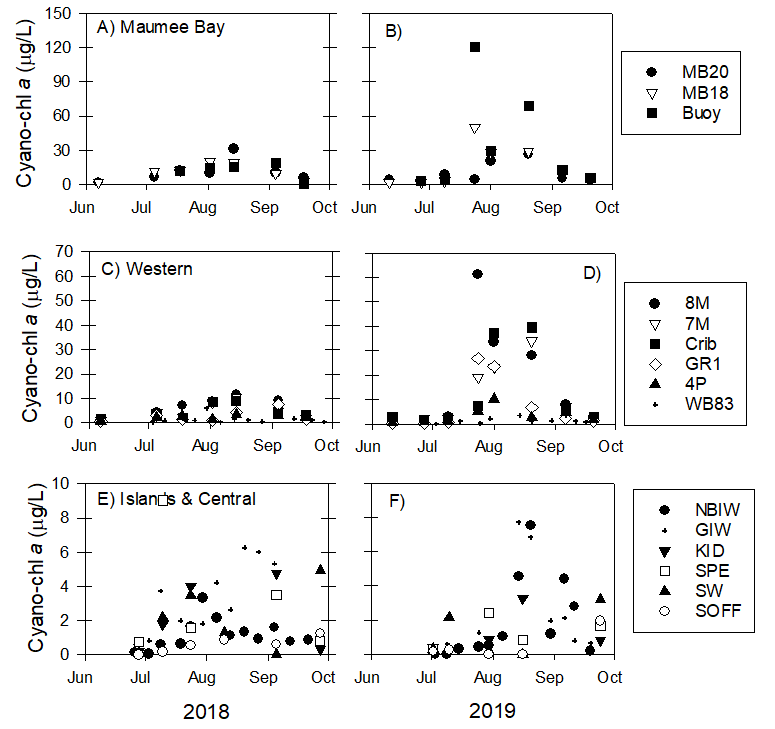


Supplemental Figure 1. Cyanobacteria-specific chlorophyll *a* concentrations at 15 sites in Lake Erie. The panels show data for sites in Maumee Bay (A, B), western Lake Erie (C, D), and around the islands on the border of the western and central basin (E, F), during the years of 2018 (left column) and 2019 (right column). Note the different scales of the Y-axes of the panels.


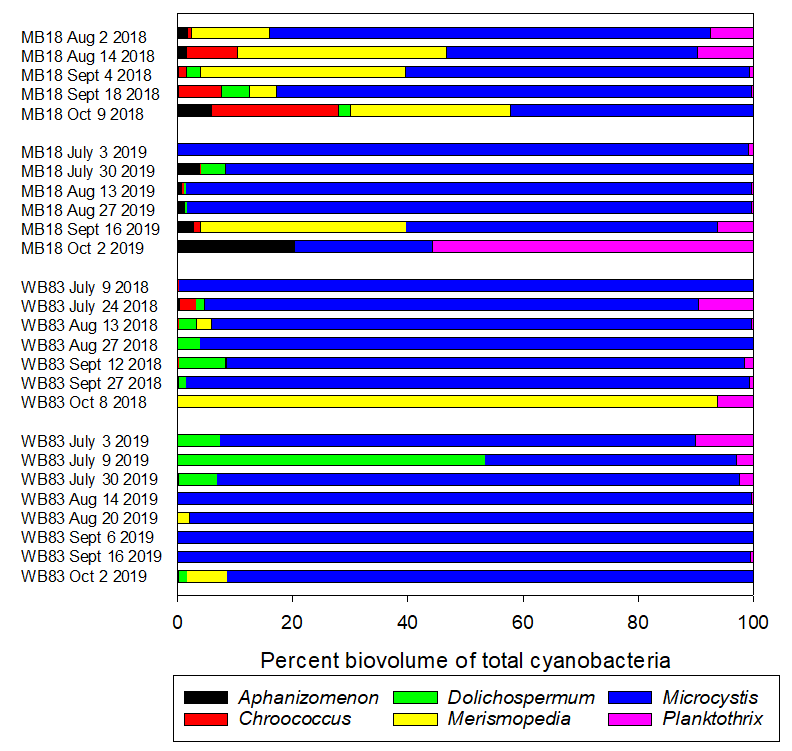


Supplemental Figure 2. The cyanobacteria community at sites MB18 and WB83 during 2018 and 2019 shown as a percent of the cyanobacteria biovolume. *Microcystis* accounted for greater than 50% in most samples, expect in October. Cyanobacteria biovolume was measured by a FlowCam 8410 in auto image mode at 100X magnification. Images were classified using a library in Visual Spreadsheet (#5.7.19) and then manually checked and reclassified as needed. Biovolume was calculated by multiplying the particles/mL by the average biovolume of the taxa, and we used the taxa shapes as suggested by Hrycik et al. (2019).


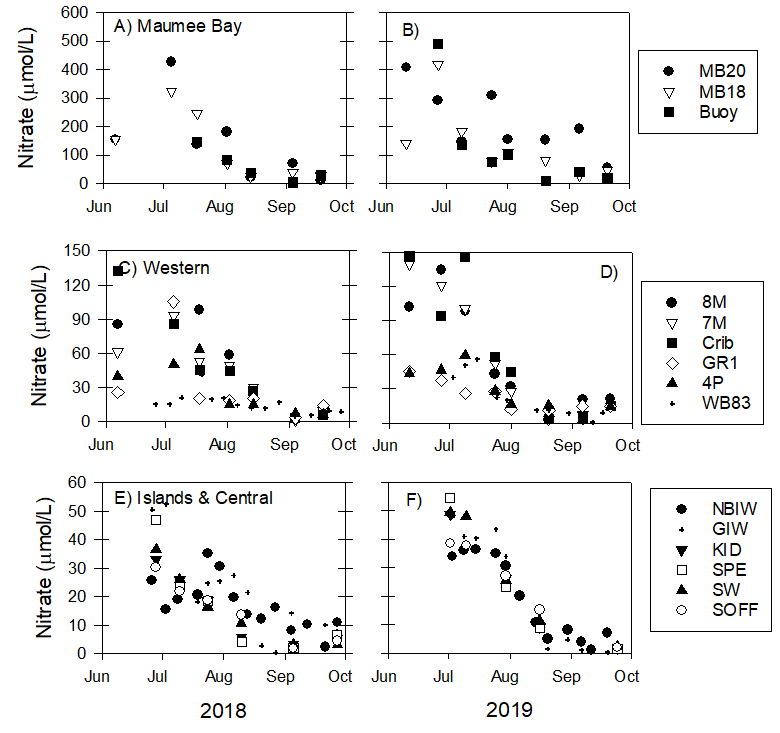


Supplemental Figure 3. Nitrate concentrations at 15 sites in Lake Erie. The panels show data for sites in Maumee Bay (A, B), western Lake Erie (C, D), and around the islands on the border of the western and central basin (E, F), during the years of 2018 (left column) and 2019 (right column). Note the different scales of the Y-axes of the panels.


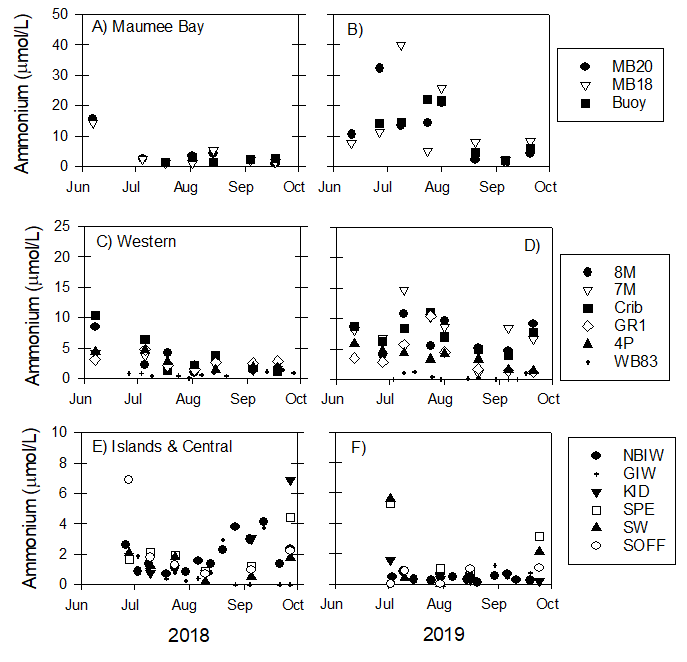


Supplemental Figure 4. Ammonium concentrations at 15 sites in Lake Erie. The panels show data for sites in Maumee Bay (A, B), western Lake Erie (C, D), and around the islands on the border of the western and central basin (E, F), during the years of 2018 (left column) and 2019 (right column). Note the different scales of the Y-axes of the panels.


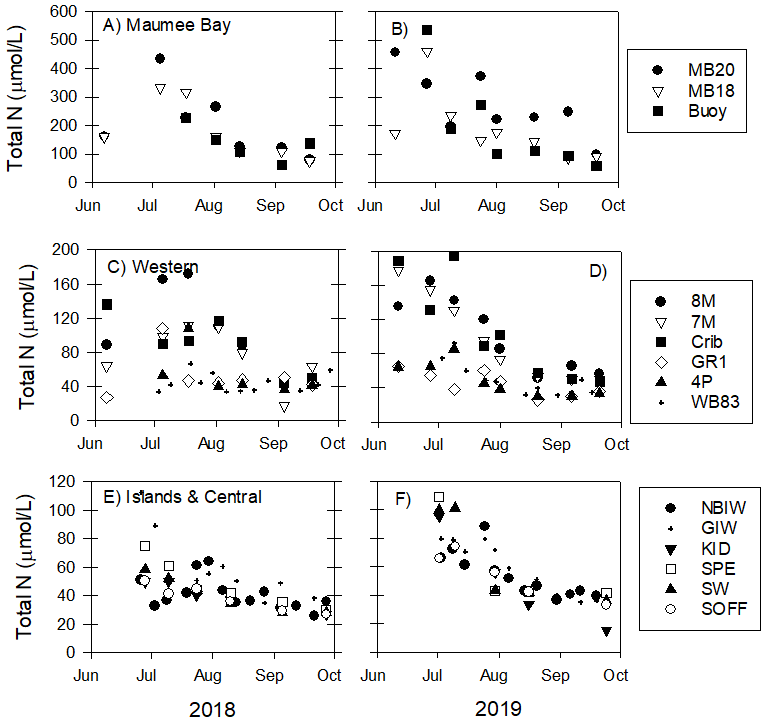


Supplemental Figure 5. Total nitrogen concentrations at 15 sites in Lake Erie. The panels show data for sites in Maumee Bay (A, B), western Lake Erie (C, D), and around the islands on the border of the western and central basin (E, F), during the years of 2018 (left column) and 2019 (right column). Note the different scales of the Y-axes of the panels.


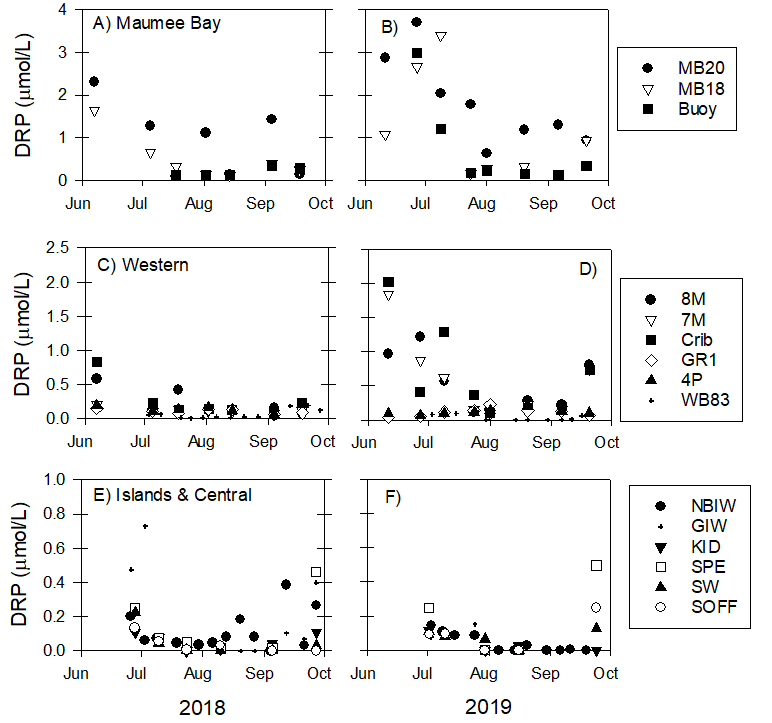


Supplemental Figure 6. Dissolved reactive phosphorus concentrations at 15 sites in Lake Erie. The panels show data for sites in Maumee Bay (A, B), western Lake Erie (C, D), and around the islands on the border of the western and central basin (E, F), during the years of 2018 (left column) and 2019 (right column). Note the different scales of the Y-axes of the panels.


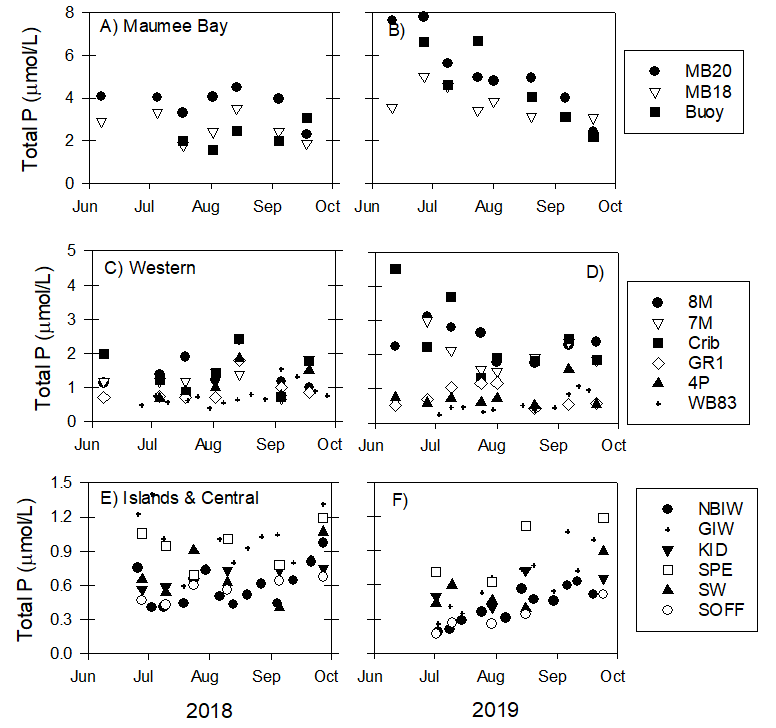


Supplemental Figure 7. Total phosphorus concentrations at 15 sites in Lake Erie. The panels show data for sites in Maumee Bay (A, B), western Lake Erie (C, D), and around the islands on the border of the western and central basin (E, F), during the years of 2018 (left column) and 2019 (right column). Note the different scales of the Y-axes of the panels.


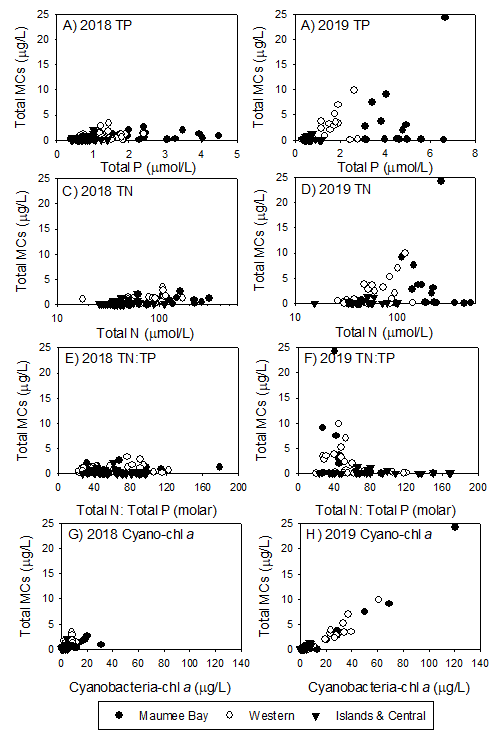


Supplemental Figure 8. Total microcystin concentrations in Lake Erie during 2018 (left) and (2019) as a function of total phosphorus (A, B), total nitrogen (C, D), the ratio of total nitrogen to total phosphorus (E, F), and cyanobacteria-specific chlorophyll *a* concentration (G, H).


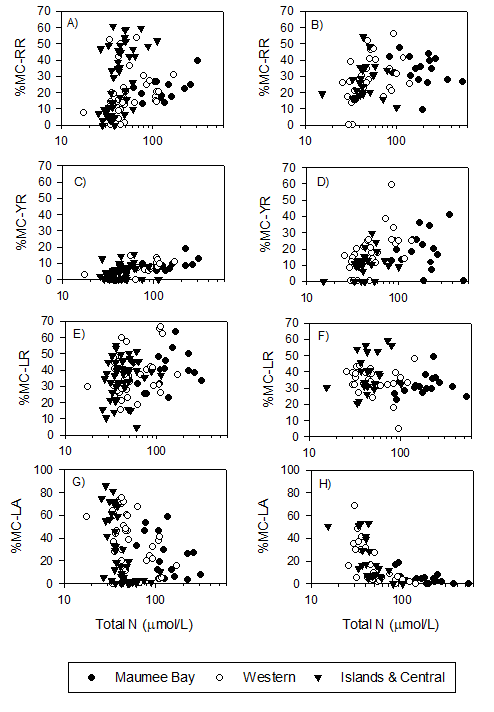


Supplemental Figure 9. Microcystin congeners in Lake Erie as the percent of total microcystins as a function of total nitrogen concentration.


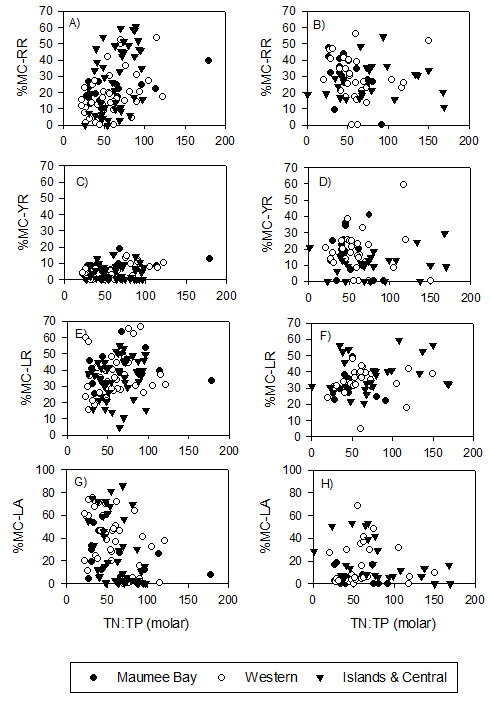


Supplemental Figure 10. Microcystin congeners in Lake Erie as the percent of total microcystins as a function of the total nitrogen to phosphorus concentration ratio.


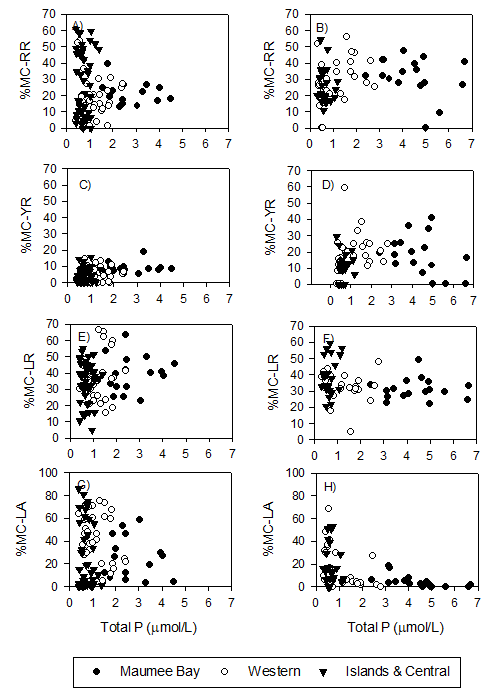


Supplemental Figure 11. Microcystin congeners in Lake Erie as the percent of total microcystins as a function of the total phosphorus concentration.


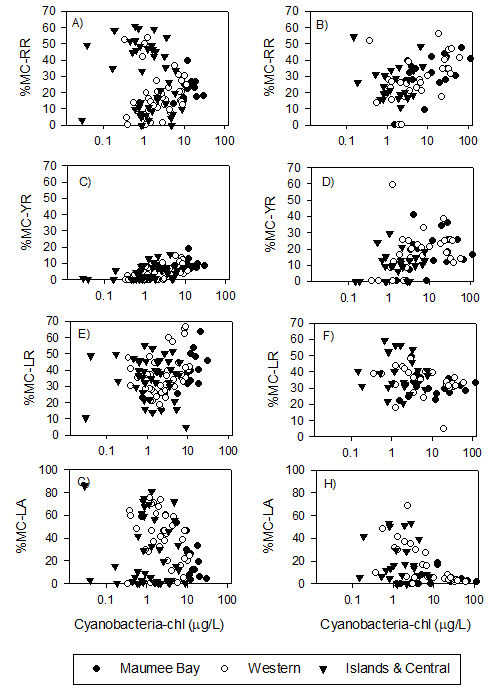


Supplemental Figure 12. Microcystin congeners in Lake Erie as the percent of total microcystins as a function of the cyanobacteria-specific chlorophyll concentration as measured by a FluoroProbe.


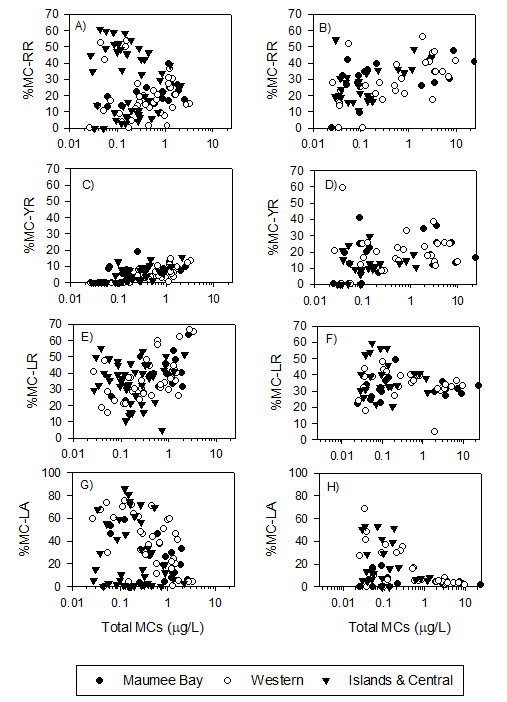


Supplemental Figure 13. Microcystin congeners Lake Erie as the percent of total microcystins as a function of the total microcystin concentration as measured by LC-MS/MS.


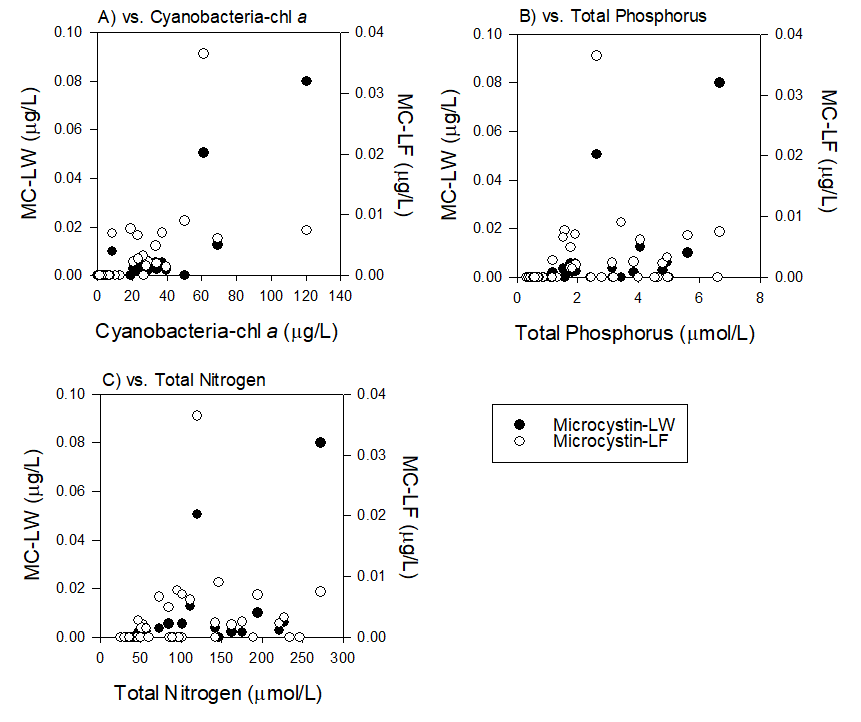


Supplemental Figure 14. Concentrations of the highly toxic microcystins, microcystin-LW (filled circles) and microcystin-LF (open circles), measured in Lake Erie during 2019 as a function of cyanobacteria-chlorophyll *a* (A), total phosphorus (B), and total nitrogen (C) concentrations.


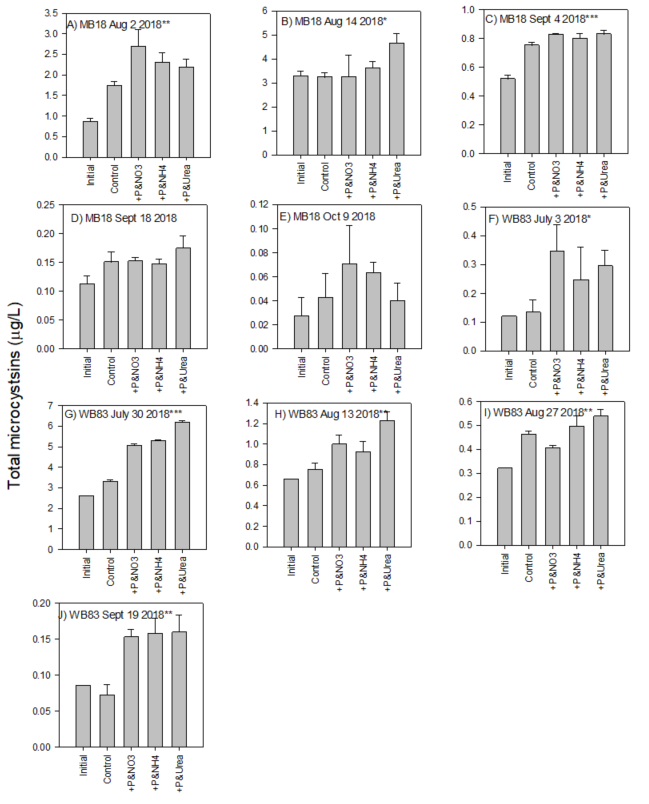


Supplemental Figure 15. Initial and final total MC concentrations in the 2018 experiments.

* = p < 0.05, ** p < 0.01, *** p < 0.001.


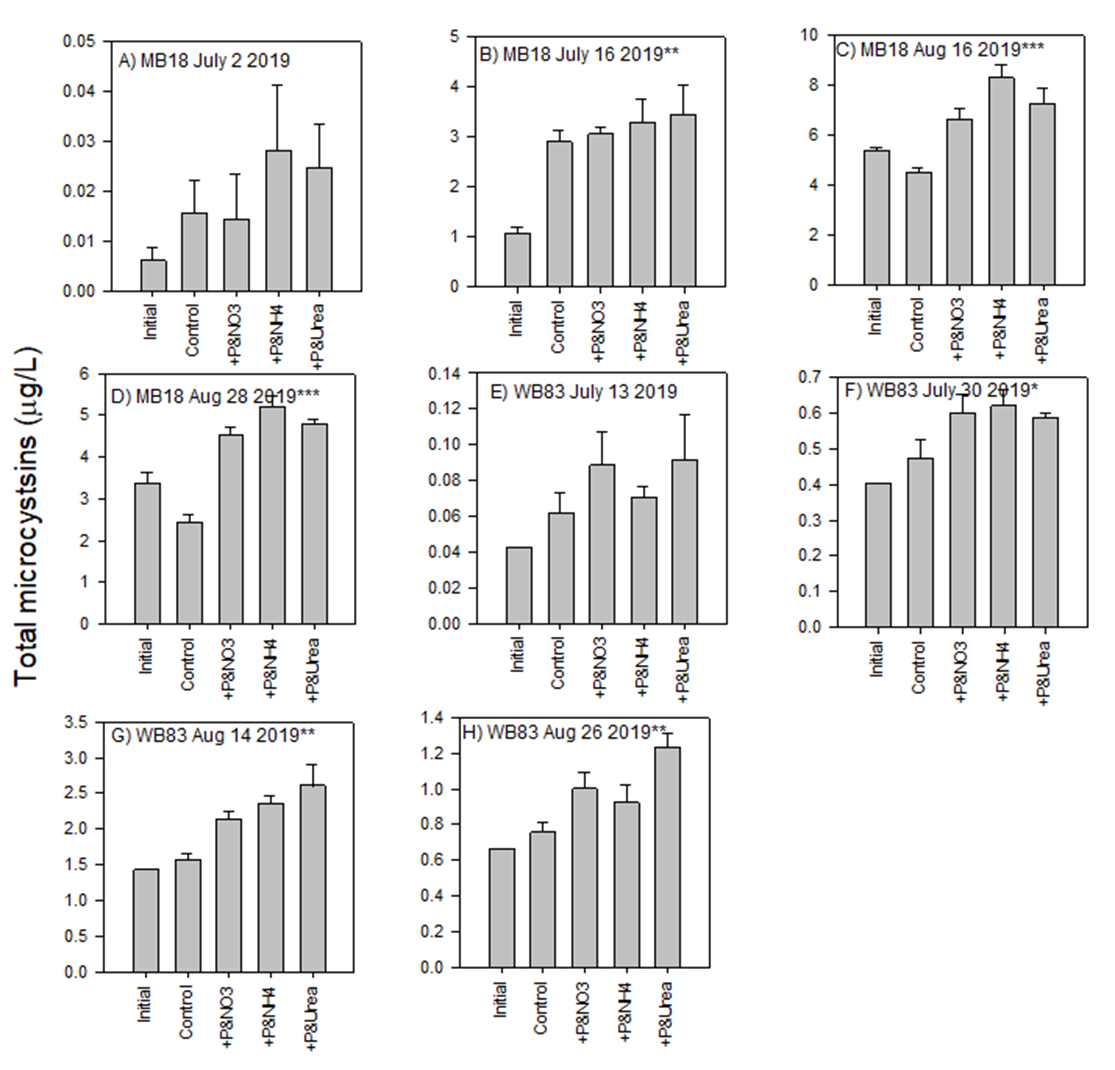


Supplemental Figure 16. Initial and final total MC concentrations in the 2019 experiments.

* = p < 0.05, ** p < 0.01, *** p < 0.001.


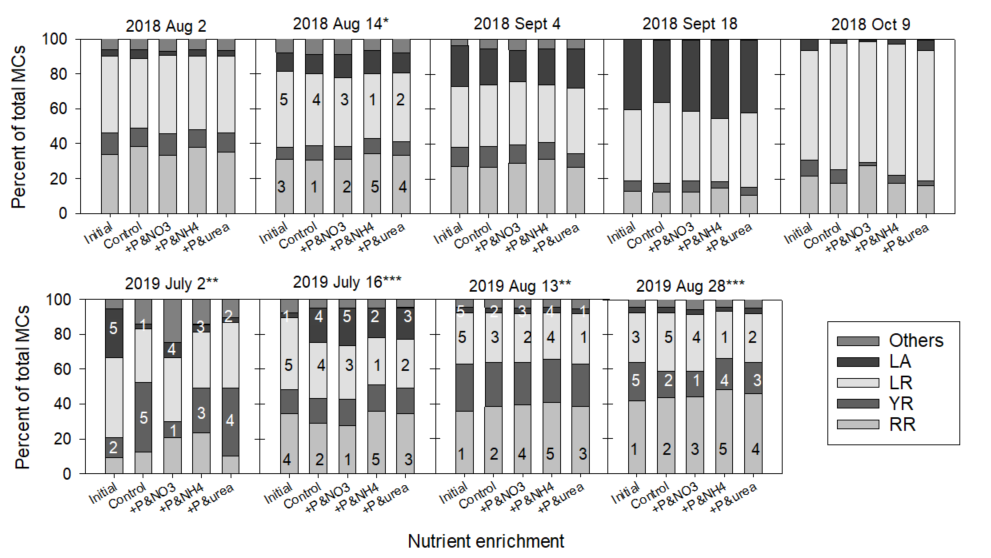
Supplemental figure 17. Microcystin congeners as percent of total in nine nutrient enrichment bioassays with water from MB18 during 2018 and 2018. Tukey test rank is superimposed onto the graph (mean of 5 > 4 > 3 > 2 >1) when the MANOVA was significant. *: p< 0.05; **: p< 0.01; ***: p< 0.001.


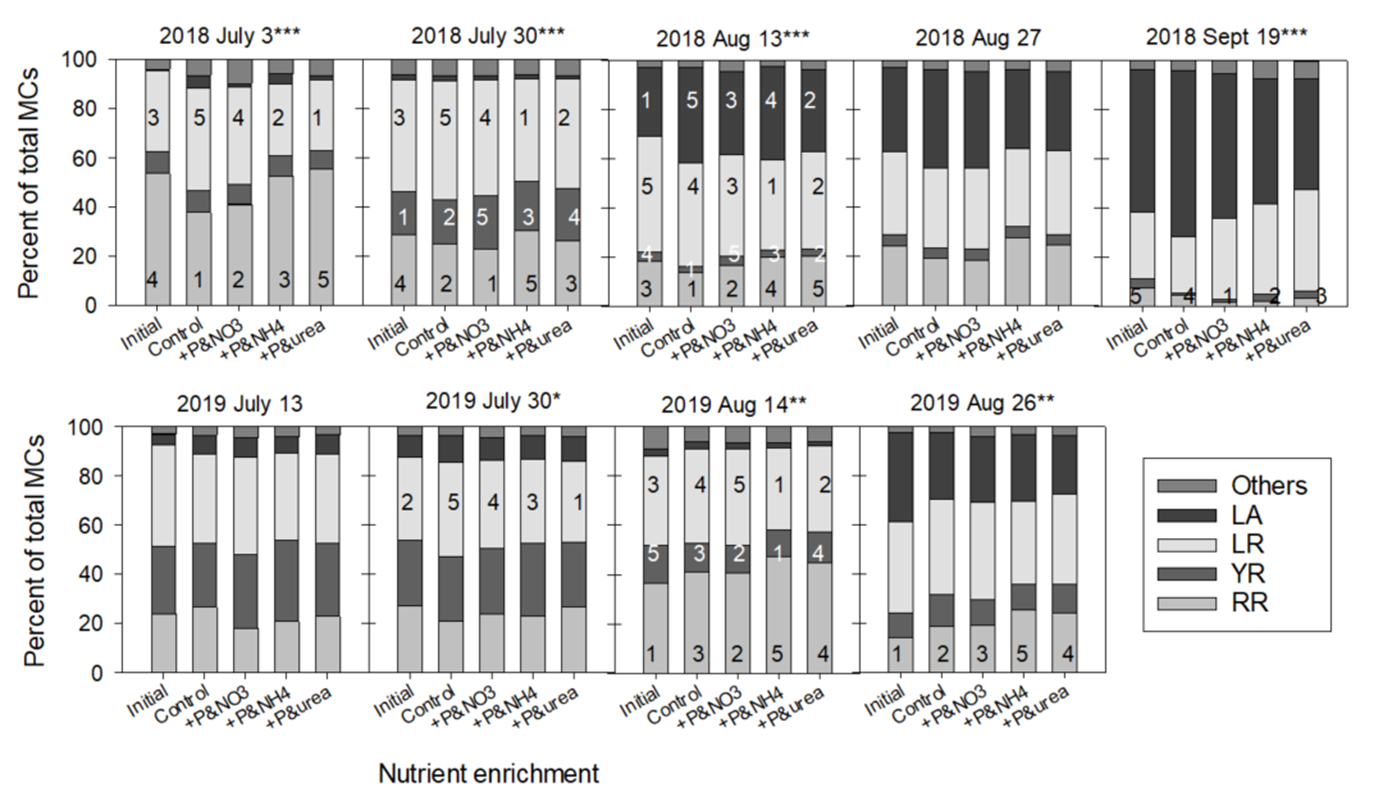


Supplemental figure 18. Microcystin congeners as percent of total in nine nutrient enrichment bioassays with water from WB83 during 2018 and 2019. Tukey test rank is superimposed onto the graph (mean of 5 > 4 > 3 > 2 >1) when the MANOVA was significant. *: p< 0.05; **: p< 0.01; ***: p< 0.001.
